# Design and synthesis of novel pyridopyrimidine moieties linked to coumarin ring of potentially active cytotoxic agents and their docking study

**DOI:** 10.1038/s41598-025-05325-1

**Published:** 2025-06-20

**Authors:** Nehal A. E. Youssef, Yasser A. Selim, Lina A. Shaheen, Ahmed A. Fadda, Hatem E. Gaffer, Sherihan A. El-Hadidy

**Affiliations:** 1 Department of Chemistry, Faculty of Science, Mansoura University, Mansoura, 35516 Egypt; 2 Faculty of Specific Education, Zagazig University, Zagazig, 44519 Egypt; 3 Faculty of Pharmacy, The British University in Egypt, Cairo, Egypt; 4 Department of Dyeing, Printing, and Auxiliaries, Textile Institute, Cairo, Egypt; 5 Department of Engineering Chemistry, Canal High Institute of Engineering and Technology, Sues, Egypt

**Keywords:** Cyclic ketone, Coumarin, Pyridopyrimidine, Cytotoxicity, Molecular Docking, Medicinal chemistry, Organic chemistry

## Abstract

Many pyridopyrimidine moieties linked to the coumarin ring were synthesized by the reaction of malononitrile with 7-hydroxy-4-methyl-2-oxo-*2 H*-chromene-8-carbaldehyde **(2)** directly in the presence of ammonium acetate and different ketones and studied the effect of other basic catalysis, ratio of reactants and the effect of the solvent. The newly synthesized compounds were evaluated for their cytotoxic activity against four cell lines namely HepG2, WI-38, VERO, and MCF-7. The cytotoxic activity showed that compounds **8**, **9**, **10**, and **7** have the highest activity against the studied cell lines. Focusing on the binding affinity and interactions between the five synthesized derivatives with the highest anticancer activity and specific amino acids of **4HJO** residues over the molecular docking analysis. Derivative **10** recorded the highest energy score with good RMSD compared to the rest of the derivatives.

**Supplementary Information:**

The online version contains supplementary material available at 10.1038/s41598-025-05325-1.

## Introduction

One of the special scaffolds, heterocycles chromene (benzopyran), is found in many natural products as a crucial structural element and also has beneficial photochemical qualities^1,2^. It has been acknowledged that chromene offers special structural advantages for the creation of novel drugs. Many biological activities are determined by the chromene scaffold’s substitution pattern. The kind, quantity, and arrangement of substituents attached to the chromene core are crucial factors in pharmacological activity^3^. The majority of compounds containing the chromene scaffold are found in natural products and exhibit a broad range of biological functions. The extensive pharmacological effects along with their low toxicity have motivated medicinal chemists to find new therapeutic drugs^4–6^. A wide range of biological activities, including antitumor^7,8^, anti-inflammatory^9^, diuretic^10^, anticoagulant^11^, antispasmolytic^8^, estrogenic^12^, antiviral^13^, antifungal^14^, antimicrobial^15^, and anti-helminthic^16^, can be attributed to the derivatives of the benzopyran moiety. Their wide range of pharmacological effects, including antibacterial, anticancer, antidiabetic, antiallergic, and anti-inflammatory effects, are well documented^17^.

However, because of its beneficial biological and pharmacological properties, including those of a diuretic, antibacterial, anticoagulant, spasmolytic, anticancer, hypnotic, and insecticide, pyran derivatives have been widely used as drug intermediates^18^. In addition, pyranopyrazoles are special heterocyclic scaffolds with several significant biological characteristics^19^.

Statistics indicate that more than 85% of physiologically active compounds are either heterocyclic or contain heterocyclic structures, with nitrogen-containing heterocycles being the most prevalent and often serving as the structural backbone of these complex molecules. The significance of heterocycles in contemporary medication design and discovery is revealed and emphasized by these facts^20^. Because of their importance in biology, chemistry, and practical applications, N-heterocycles are vital. In biological processes involving antitumor, antiviral, antibacterial, anti-inflammatory, anticancer, and antidiabetic investigations, they are extensively studied^21^. Scientists have been interested in nitrogen-containing heterocycles for a long time because of their biological significance and diversity of structures, which make them promising candidates for cancer chemotherapy^22^.

Over the past 60 years, pyrimidines have grown in importance as a fundamental structural component of many therapeutic compounds. This article reviews the most current areas such as anti-infectives, anticancer, immunology, immuno-oncology, neurological disorders, chronic pain, and diabetes mellitus where pyrimidines have significantly influenced drug discovery treatments^23^.

Pyrimidine derivatives represent a significant class of compounds known for their diverse pharmacological properties, including antiviral, anticancer, antibacterial, and antihypertensive activities^24^. Aza-analogs of 1,4-dihydro pyridines, such as dihydropyrimidines (DHPMs), have garnered attention in recent times due to their comparable pharmacological profile to that of traditional dihydropyridine calcium channel modulators^25–30^.

The dihydropyrimidines are well known for their ability to inhibit calcium channels, but they are also being investigated for potential therapeutic benefits in the treatment of AIDS^31^. This is because the natural marine alkaloids batzelladine A and B, which are the first low molecular weight natural products reported in the literature to inhibit the binding of HIV gp-120 to CD4 cells, have been found to possess their specific structure^31^. This opens up a new avenue for the development of AIDS therapy.

## Results and discussion

**Fig. 1:**
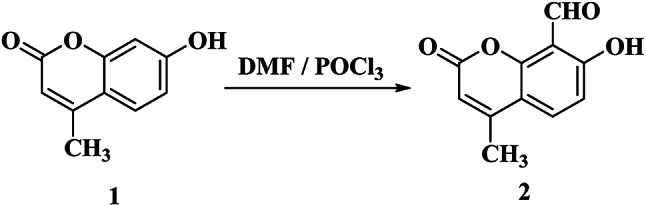
Synthesis of compound **2**.

Many investigations have been reported for the formylation of 7-hydroxy-4-methylcoumarin **(1)** using hexamine in boiling acetic acid for a long time, the yield was usually low, and many problems are found in the purification of the product. Panel et al.^32^ described a procedure to yield compound **2** in 60% yield. These problems prompted us to study the formylation reaction using Vilsmeier Haack reagent which afforded compound **2** with a yield of around 70%. The structure of compound **2** was established by physicochemical and literature data^32^ (Fig. 1).

The utility of activated nitriles in the synthesis of heterocyclic compounds has been investigated recently in our lab numerous reports have been appeared also in the literature which highlight the chemistry of the activated nitriles and their uses as intermediates in organic chemistry^33–35^.

In the present work, we report here the reaction of compound **2** with different ratios of malononitrile with and without different ketones in the presence of ammonium acetate to give chromenopyrimidine derivatives while establishing their chemical and biological activities.

**Fig. 2:**
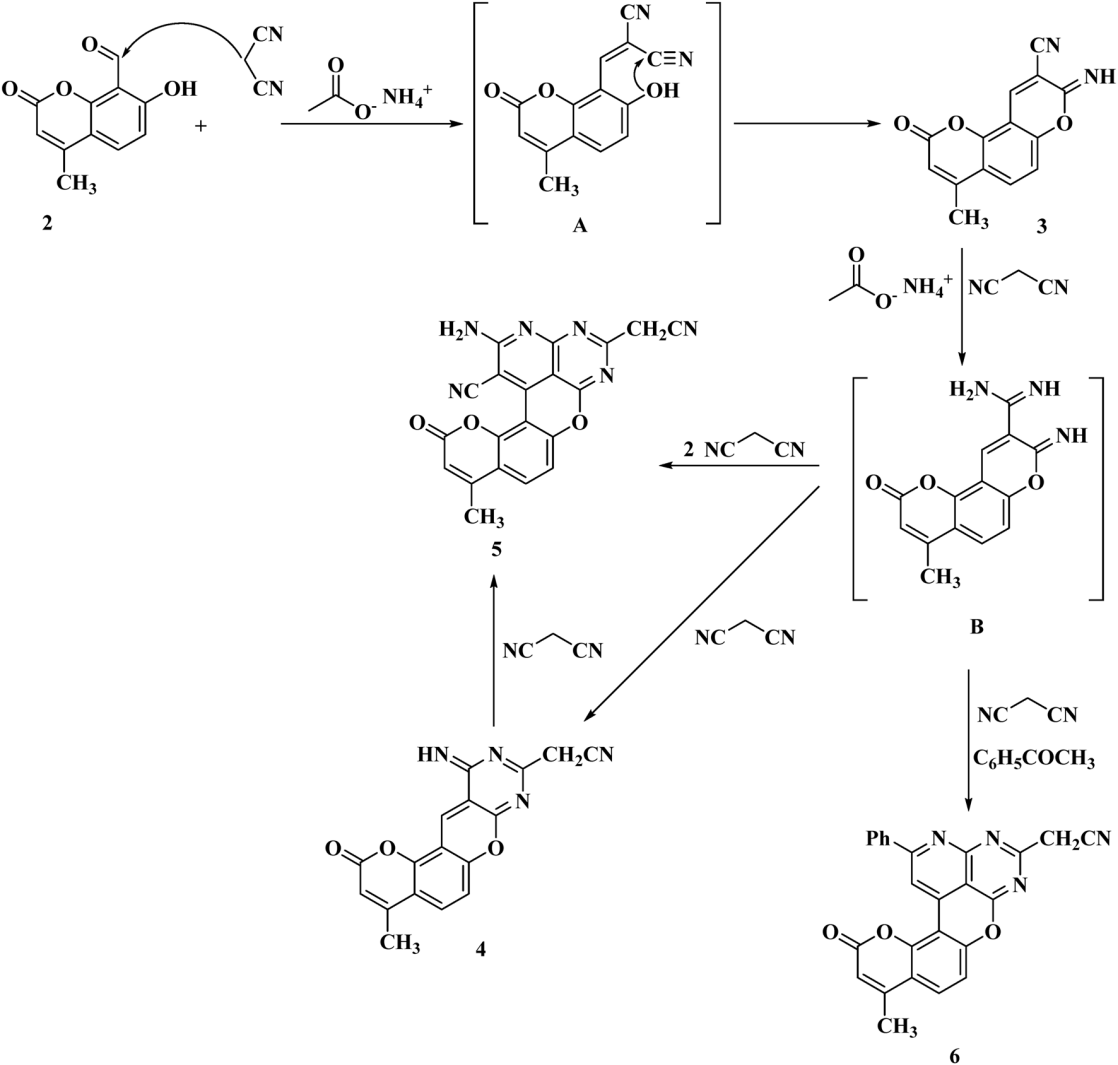
Reaction of compound **2** with malononitrile.

Thus, condensation of compound **2** with malononitrile in equimolar ratio in ethanol and in the presence of a slight excess of ammonium acetate afforded 8-imino-4-methyl-2-oxo-*2 H*,*8 H*-pyrano[2,3-*f*]chromene-9-carbonitrile **(3)** (Fig. 2). The IR spectrum of compound **3** showed the presence of absorptions at *ν* 3250, 2220, 1733, and 1620 cm^− 1^ due to NH, CN, CO, and C = N, respectively. The ^1^H-NMR spectrum of compound **3** showed peaks at δ 2.42 ppm singlet signal due to CH_3_ protons, singlet signal at δ 6.18 ppm due to C_3_-H proton, doublet at δ 6.97 ppm, *J* = 9.20 Hz, C_5_-H proton, doublet at δ 7.59 ppm, *J* = 9 Hz, C_6_-H proton, singlet signal at δ 8.71 ppm for C_10_-H proton, and finally singlet signal at δ 9.36 ppm for the NH proton (Fig. S2).

In this case, the Knoevenagel condensation of compound **2** on malononitrile afforded the intermediate **A**. However, the intra-molecular addition of OH to the cyano group afforded the final product **3**.

When compound **2** was heated with an excess of malononitrile in ethanol, 2-amino-5-(cyanomethyl)-10-methyl-12-oxo-*12 H*-7,13-dioxa-3,4,6-triazabenzo[*no*]tetraphene-1- carbonitrile **(5)** was obtained as the only main product. On the other hand, when the reaction was carried out in the presence of a catalytic amount of triethylamine or piperidine instead of ammonium acetate, compound **3** was obtained as the main product.

It seems reasonable to assume that the formation of compound **4** may be achieved by the following process. In such a mechanism, malononitrile is firstly condensed with compound **2** to give compound **3** which in turn is converted to 8-imino-4-methyl-2-oxo-*2 H*,*8 H*-pyrano[2,3-*f*]chromene-9-carboximidamide **(B)** by the addition of a molecule of ammonia to the cyano group. Moreover, the intermediate **B** condenses with the second molecule of malononitrile to give 2-(11-imino-4-methyl-2-oxo-*2 H*,*11 H*-pyrano[2’,3’:5,6]chromeno[2,3-*d*]pyrimidin-9-yl)acetonitrile **(4)**, which further reacts with another molecule of malononitrile yielding finally compound **5 ** (Fig. 2).

All attempts to control the reaction to afford the formation of **(B)** were unsuccessful. Even when limited quantities of malononitriles were used under conditions favorable for monocyclo addition, the product was almost **3**. The infrared spectrum of compound **4** showed bands at *ν* 3355 (NH), 2218 (CN), 1737 (C = O), and 1620 cm^− 1^ (C = N) stretching vibration, respectively. The ^1^H-NMR of **4** showed singlet signal at δ 2.16 ppm due to CH_3_ protons, 3.15 ppm singlet signal for CH_2_CN protons, two singlet signals at δ 6.05 and 7.81 ppm corresponding to C_3_-H, and C_10_-H, respectively, two doublets’ signals at δ 6.84 and 7.45 ppm due to C_6_-H, and C_5_-H, respectively, and finally singlet signal at δ 11.90 ppm for NH proton (Fig. S4). On the other hand, the ^1^H-NMR of compound **5** displayed a singlet signal at δ 2.43 ppm corresponding to CH_3_ protons, a singlet signal at δ 4.10 ppm due to CH_2_CN protons, a singlet signal at δ 6.30 ppm for C_3_-H cumarine, a singlet signal of NH_2_ protons at δ 6.92 ppm, doublet at δ 7.21 ppm, *J* = 9.1 Hz, for C_8_-H, and finally doublet at δ 7.93 ppm corresponding to C_9_-H (Fig. S6).

Heating of a mixture of malononitrile, acetophenone, and compound **2** in equimolar ratio for two hours in the presence of excess ammonium acetate afforded a mixture of compound **4** (16%), compound **5** (12%), and (25%) of 2-(10-methyl-12-oxo-2-phenyl-*12 H*-7,13-dioxa-3,4,6-triazabenzo[*no*]tetraphen-5-yl)acetonitrile **(6)** (Fig. 2).

Minimizing reflux time to only 10 min afforded a mixture containing compounds **3** (18%), compound **4** (12%), and compound **6** (10%), while compound **5** could not be detected, all attempts to limit this reaction to the 1:1 adduct such as using limited amounts of malononitrile under conditions favorable for monocyclo addition were unsuccessful. On the other hand, when this reaction was refluxed for a short or long time in the presence of triethylamine, compound **3** was formed as the only main product.

The IR spectrum of compound **6** showed absorption bands at *ν* 2218 and 1620 − 1500 cm^–1^ attributable to stretching vibration, in generally, to C = N of hetero ring and C = C specially in phenyl group, respectively, meanwhile, this spectrum did not show absorption in the amino or imino regions. This fact is taken to indicate that in many cases in the present study, the course of the reaction was markedly influenced by the choice of the base catalyst and/or the reaction time. In this reaction, malononitrile is firstly condensed with compound **2** to give **3**, which in turn is converted to amidinoiminochromene intermediate **A**. The later intermediate condenses with the carbonyl of the ketone, cyclization between the methyl group of ketones and the 4-position of the coumarin ring, and subsequent dehydrogenation takes place, finally giving compound **6 ** (Fig. 2). The ^1^H-NMR of compound **6** showed peaks at δ 2.26 ppm singlet signal of CH_3_ protons, singlet signal at δ 3.88 ppm due to CH_2_CN, singlet signal at δ 6.18 ppm due to C_11_-H, two doublet signals at δ 6.99 and 8.40 ppm due to C_8_-H and C_9_-H, respectively, singlet signal at δ 7.80 ppm due to C_10_-H pyrane, while the aromatic protons appeared as multiplet signals in the region at 7.22–7.41 ppm (Fig. S7).

**Fig. 3:**
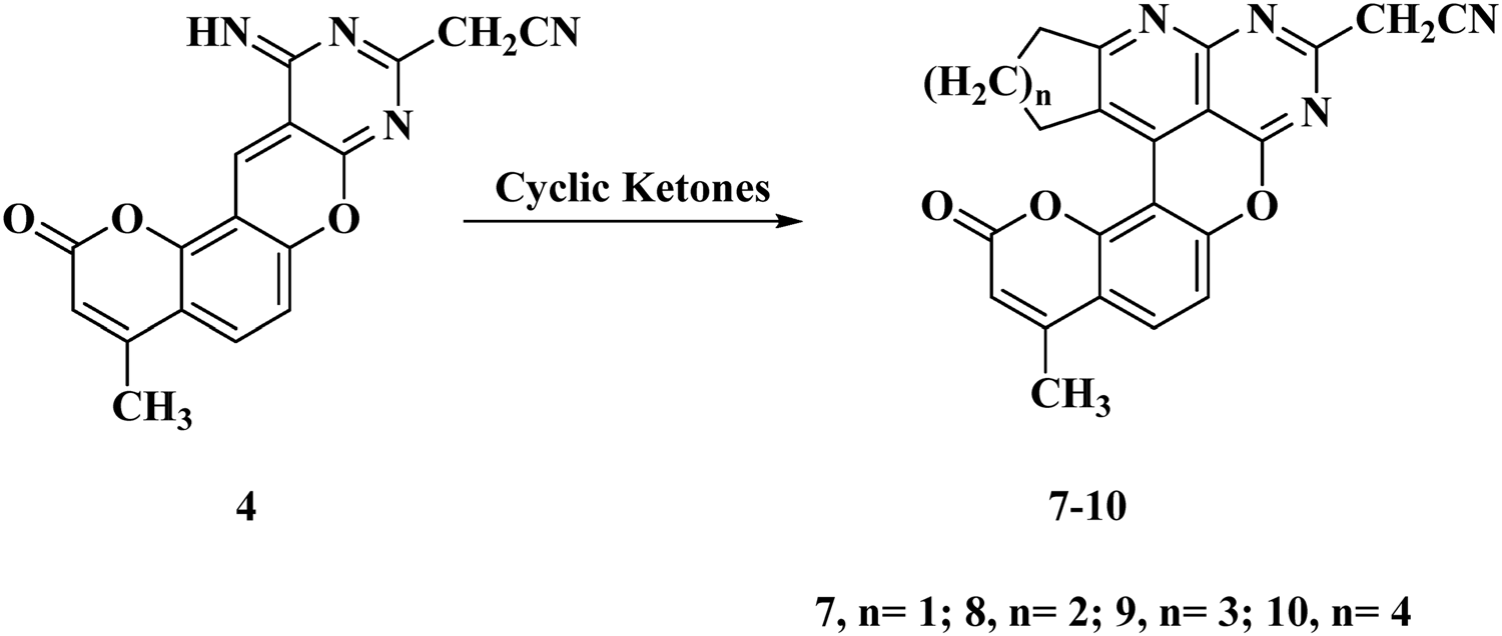
Reaction of compound **4** with cyclic ketones.

Moreover, the reaction with cyclic ketone proceeded much as the case of the reaction of the aromatic ketone described above. In this case, cyclization occurred between the methylene group adjacent to the carbonyl group of the ketone and the activated double bond in compound **4** followed by cyclocondensation of the produced Michael adduct to the final products **7–10** with the elimination of water (Fig. 3). The IR spectra, in general, showed no absorption bands for amino or imino groups. In general, compounds **7–10** showed in their IR spectra absorption bands at *ν* 2220, and 1612 cm^− 1^ attributable to the cyano and C = N groups, respectively. The ^1^H-NMR of compound **7** showed a multiplet signal at δ 2.11–2.30 ppm for the CH_2_ group, a singlet signal at δ 2.32 ppm for CH_3_ protons, triplet at δ 3.00 ppm due to the CH_2_ group, triplet at δ 3.11 ppm for CH_2_ protons, a singlet signal at δ 3.79 ppm for CH_2_-CN, 6.25 singlet for C_3_-H coumarin, 7.15 and 7.70 ppm two doublets for aromatic protons (Fig. S8).

**Fig. 4:**
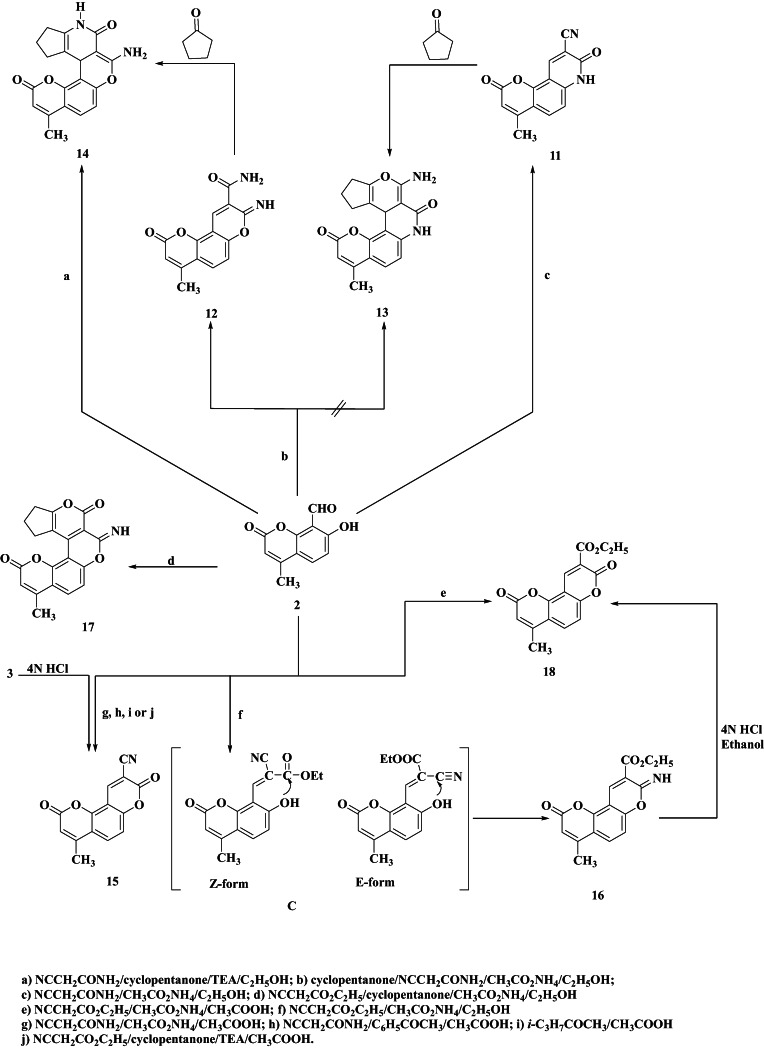
Reaction of compound **2** with various reagents.

Previously reported work has shown that, cyanoacetamide condensed with ketones^36^ or aldehydes^37,38^ in the presence of ammonium acetate to give substituted pyridone, quinolines, and benzoquinozolines. The present work will deal with the study of the behavior of cyanoacetamide and ethyl cyanoacetate on condensation with compound **2**. The reactions are outlined in Fig. 4. It was found that compound **2** reacts with cyanoacetamide instead of malononitrile in the presence of ammonium acetate in absolute ethanol to yield 78% of compound **11** as a single product (TLC control) (Fig. 4).

The IR spectrum of **11** showed absorption bands at *ν* 1675, 2218, and 3335 cm^− 1^ attributable to amidic carbonyl, cyano, and NH groups, respectively. Using TEA instead of ammonium acetate in the above-mentioned reaction as a basic medium afforded a mixture of **10** and **12**. Unexpectedly, when compound **2** was allowed to react with a mixture of cyanoacetamide and cyclopentanone in absolute ethanol and in the presence of ammonium acetate, it gave the 8-imino-4-methyl-2-oxo-*2 H*,*8 H*-pyrano[2,3-*f*]chromene-9-carboxamide **(12)** instead of the corresponding 2-amino-7-methyl-*10c*,11,12,13-tetrahydro-*3 H*-cyclopenta[5,6]pyrano[3,4-*c*] pyrano[2,3-*f*]quinoline-3,9(*4 H*)-dione **(13)** in good yield. Using TEA instead of ammonium acetate as a catalyst in the above-mentioned reaction afforded two isolable products, **(13)** and its isomeric product **(14)**. The formation of these products is assumed to proceed *via* the addition of the cyclopentanone C-2 carbon to the activated double bond in **11** and **12** (which is assumed to be formed first) followed by cyclization of the produced Michael adduct to give the required products **13** and **14**, respectively. The structures of **13** and **14** were established not only on the basis of their analytical and spectral data but also on their independent synthesis *via* the reaction of either **11** or **12** with cyclopentanone and ammonium acetate in absolute ethanol to give the corresponding **13** and/or **14**, respectively (Fig. 4). The IR spectra of **13** and **14** revealed absorption bands near 1630 − 1500, 1680, and 3250 cm^–1^ characteristics of stretching vibrations of the hetero ring, amidic carbonyl, and NH function, respectively.

Interestingly, when compound **2**, cyanoacetamide, and ammonium acetate were allowed to react with acetophenone, cyclopentanone, and/or isopropyl methyl ketone in acetic acid the 4-methyl-2,8-dioxo-*2 H*,*8 H*-pyrano[2,3-*f*]chromene-9-carbonitrile **(15)** was obtained as the only isolable product (Fig. 4). Its IR spectrum showed two characteristic absorption bands at *ν* 1739 and 2220 cm^–1^, characteristic of carbonyl and cyano groups, respectively. The formation of **15** in such cases indicates that the use of acetic acid as a solvent did not favor any further cycloaddition reaction, and consequently the reaction was stopped at this stage of the reaction. On the other hand, when an ethanolic solution of compound **2** was allowed to react with ethyl cyanoacetate in the presence of ammonium acetate, it afforded the ethyl 8-imino-4-methyl-2-oxo-*2 H*,*8 H*-pyrano[2,3-*f*]chromene-9-carboxylate **(16)** (Fig. 4). Additionally, the Knoevenagel adduct is anticipated to occur in the active methylene of ethyl cyanoacetate as an intermediate between the two potential isomeric configurations, the Z- and E-forms (intermediate C). The main isolated product, identified by ^1^H-NMR and analytical data, was the imino coumarin **16**, which was created by the E-configured Knoevenagel adduct. In contrast to the behavior of cyclopentanone with cyanoacetamide, compound **2** reacted with ethyl cyanoacetate in the presence of ammonium acetate and ethanol to yield the cycloaddition product 3-imino-7-methyl-3,11,12,13-tetrahydro-*2 H*,*9 H*-cyclopenta[5,6]pyrano[3,4-*c*]pyrano[2,3-*f*]chromene-2,9-dione **(17)** (Fig. 4). However, when this reaction was repeated in the presence of TEA as a base, **15** was obtained as the only product. Compound **15** was synthesized independently *via* hydrolysis of **3** in a mixture of conc. hydrochloric acid and ethanol. In addition, the reaction of compound **2**, ethyl cyanoacetate, and ammonium acetate in acetic acid afforded ethyl 4-methyl-2,8-dioxo-*2 H*,*8 H*-pyrano[2,3-*f*]chromene-9-carboxylate **(18)**. Compound **18** was synthesized independently *via* hydrolysis of **16** in a mixture of conc. hydrochloric acid and ethanol (Fig. 4). The IR spectrum for this product revealed two absorption bands at *ν* 1700 and 1735 cm^–1^ characteristic of cyclic and acyclic carbonyl moieties, respectively. The procedures described in this investigation were found to be satisfactory for the synthesis of a wide variety of poly heterocyclic compounds in good yield.

### Cytotoxicity (anticancer screening)

Globally, cancer is the leading cause of death, accounting for around six million deaths annually^39^. The goal of cancer control should ultimately be prevention^40^. There are various ways to treat cancer in contemporary medicine. Among these treatments include radiation, chemotherapy, and surgery. These days, chemotherapy is the most effective cancer treatment technique. The use of chemopreventive drugs early in the carcinogenesis process appears to make more sense theoretically than the use of chemotherapeutic treatments to eradicate a fully grown tumor. Rather, most cancer chemotherapeutic medications negatively impact the majority of normal cells^41^. Finding novel anticancer drug entities that are less detrimental to the host cell is crucial^42,43^. Heterocyclic compound use proved crucial to the prevention and treatment of cancer^44^.

**Fig. 5:**
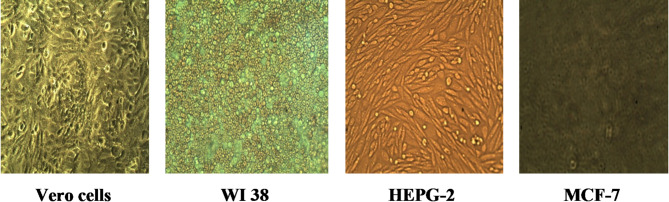
Confluent monolayers of cell lines used for testing.

Keeping the aforementioned details in mind, the objective of this study was to synthesize new pharmaceuticals for anticancer assessment as a trial to produce newer antitumor drugs with increased activity and minimal adverse effects. Human hepatocellular liver carcinoma cell line (HepG2), human lung fibroblast cell line (WI-38), human Caucasian breast adenocarcinoma cell line (MCF-7), and normal adult African green monkey kidney cell line (VERO) were the four different types of human cell lines used to test the anticancer activity of all newly synthesized compounds (Fig. 5). In regard to the pharmacological reference, the inhibitory concentration (IC_50_) is the concentration needed to prevent 50% of the growth of cells after 72 h of incubation (Table 1). Compared to the drug reference 5-fu, compounds **8**, **9**, **10**, and **7** had very significant action against HepG2 cellular carcinoma, with IC_50_ values of 4.90 ± 0.11, 5.40 ± 1.22, 5.80 ± 1.03, and 9.60 ± 1.22 µg/mL, respectively. Additionally, these substances exhibited extremely potent action against the four examined cell lines.

**Table 1:** Cytotoxicity (IC_50_) of tested compounds on different cancer cell lines.

Compound	IC_50_, µg/mL			
	HepG2	WI-38	VERO	MCF-7
**3**	59.00 ± 0.22	68.20 ± 0.07	71.02 ± 0.05	70.00 ± 0.06
**4**	24.50 ± 0.11	31.00 ± 0.01	34.10 ± 0.15	31.40 ± 0.11
**5**	21.20 ± 1.22	27.50 ± 0.31	31.20 ± 0.02	30.02 ± 1.01
**6**	19.10 ± 1.13	26.00 ± 0.21	24.10 ± 0.19	22.50 ± 0.04
**7**	9.60 ± 1.22	9.90 ± 1.04	11.50 ± 0.05	9.50 ± 0.04
**8**	4.90 ± 0.11	7.50 ± 0.01	5.70 ± 0.06	5.10 ± 0.18
**9**	5.40 ± 1.22	5.90 ± 0.03	6.60 ± 0.02	7.20 ± 1.02
**10**	5.80 ± 1.03	7.80 ± 0.1	8.20 ± 1.04	8.90 ± 1.02
**11**	32.20 ± 1.22	39.01 ± 0.07	35.50 ± 0.01	33.30 ± 1.06
**12**	37.50 ± 1.13	48.20 ± 1.04	51.30 ± 1.05	49.40 ± 0.19
**13**	45.20 ± 1.13	56.00 ± 1.14	58.20 ± 1.05	53.00 ± 0.19
**14**	49.00 ± 1.22	56.00 ± 0.32	58.00 ± 1.02	52.00 ± 0.01
**15**	35.20 ± 1.13	38.01 ± 1.22	40.30 ± 1.19	37.03 ± 0.04
**16**	52.50 ± 1.22	59.30 ± 1.04	57.50 ± 0.05	52.10 ± 0.04
**17**	40.20 ± 0.23	53.50 ± 0.1	56.20 ± 1.06	52.50 ± 0.18
**18**	54.00 ± 0.22	60.40 ± 0.03	66.50 ± 1.02	57.00 ± 1.23
**5-Flu**	2.50 ± 1.03	5.50 ± 0.0	4.00 ± 0.04	3.00 ± 0.02

IC_50_ (µg/ mL): 1–10 (very strong), 11–25 (strong), 26–50 (moderate), 51–100 (weak), 100–200 (very weak), 200 (noncytotoxicity), 5-Flu = 5-fluorouracil.

### Structure-activity- relationship (SAR)

Adenine (A), thymine (T), guanine (GUA), and cytosine (C) are the four types of nucleotides that make up DNA. While thymine and adenine are almost coupled, cytosine and guanine are coupled *via* hydrogen bonds^45,46^. When examining the cytotoxicity of synthetic compounds tested on various cell lines, two variables might be deemed reliable:

1. the creation of an intramolecular hydrogen bond with any DNA nucleotide.
2. The positive charge on the tested compounds and the negative charge on the cell wall are attracted to one other.

The following observations were made regarding the SAR:

- Compounds **8**, **9**, **10**, and **7** revealed very strong activity against the four tested cell lines, let us consider several key factors that contribute to their cytotoxicity. These include structural feathers, electronic effects, lipophilicity, and any available data on their biological activity.
- Compound **8** is a highly complex structure with multiple heteroatoms which can enhance binding affinity to biological targets. Moreover, cyanomethyl group can increase the potential for interactions with cellular targets.
- Compound **9** Similar to **8** with an additional ring, possibly increasing the potential interactions with biological targets. Compound **10** is also another highly complex structure, similar to **8** and **9**, indicating potential for strong cytotoxic activity.
- Compound **7** is slightly less complex than **8**, **9**, and **10**, but still retains significant structural features for potential cytotoxicity. The presence of a phenyl group in compound **6** can enhance lipophilicity and possibly improve cell membrane permeability.
- Moreover, in general, compounds containing strong electron-withdrawing carbonyl group, exhibited strong activity toward the tested cell lines. This activity caused damage to the DNA nucleobases through electrostatic attraction.
- Compounds **5** and **4** showed significant activity; this could be explained by the electron-withdrawing groups, NH and NH_2_, that are present in the compounds’ structures and can be added to DNA’s unsaturated moiety and cause damage to DNA through hydrogen bonding with one of the nucleotide bases.
- In addition to having more oxo groups, compound **15** also has pyrano-pyrimidine groups, which are strong electron-withdrawing groups that generate electrostatic interaction with DNA nucleobases and damage them.
- Compounds **11** and **12** are similar to the activity of **4** and **5**. Compound **18** has a carboxylate group that could reduce cytotoxicity compared to nitriles or amides.
- Compound **3** is a simple structure with imino and nitrile groups, less complex than other compounds.

In conclusion, all compounds have more than one oxo, cyano, or NH group in their structure, which gives it the ability to disrupt DNA by forming an intramolecular H-bond with one of their nucleobases.

### Molecular docking

**Fig. 6:**
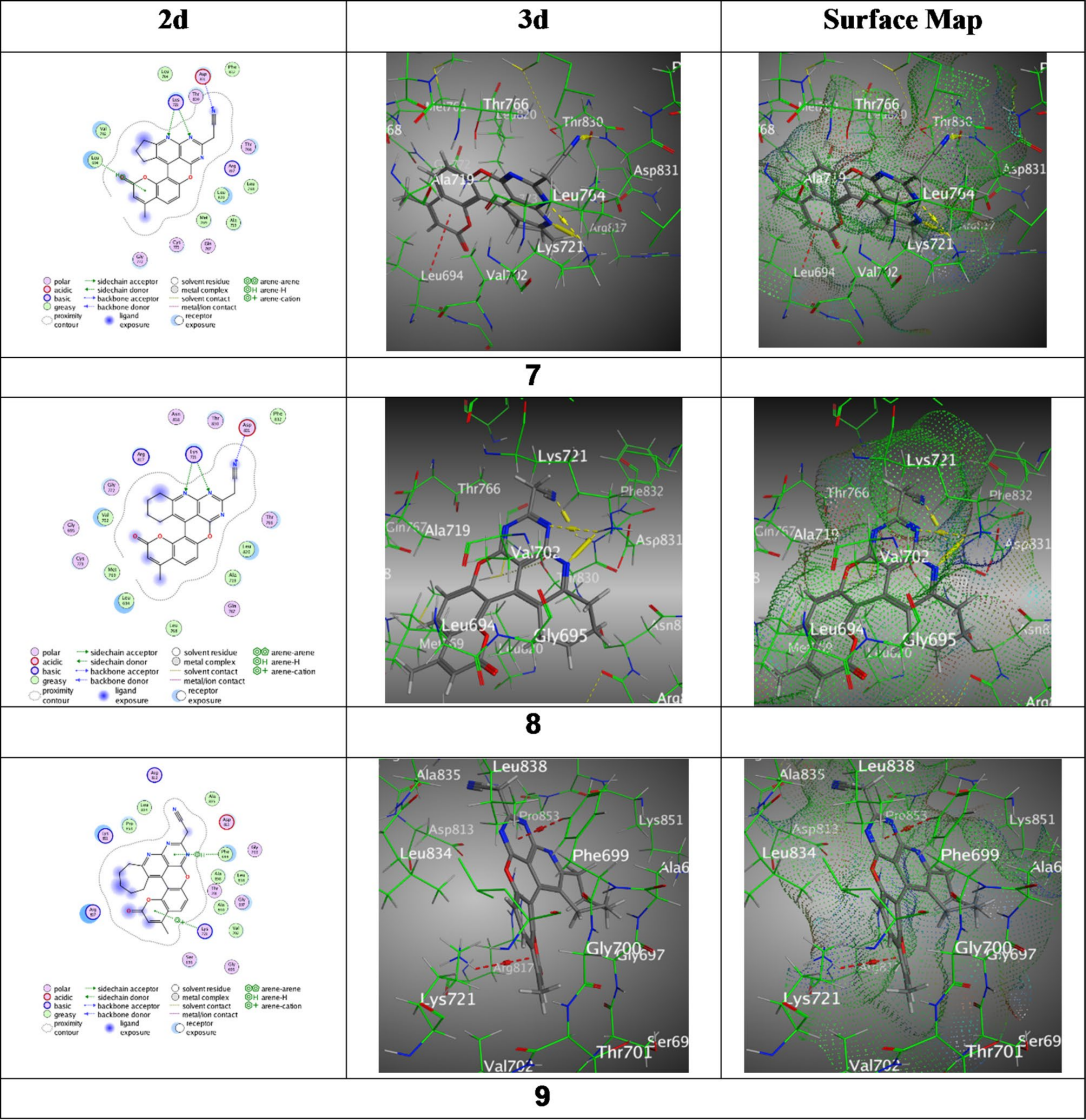

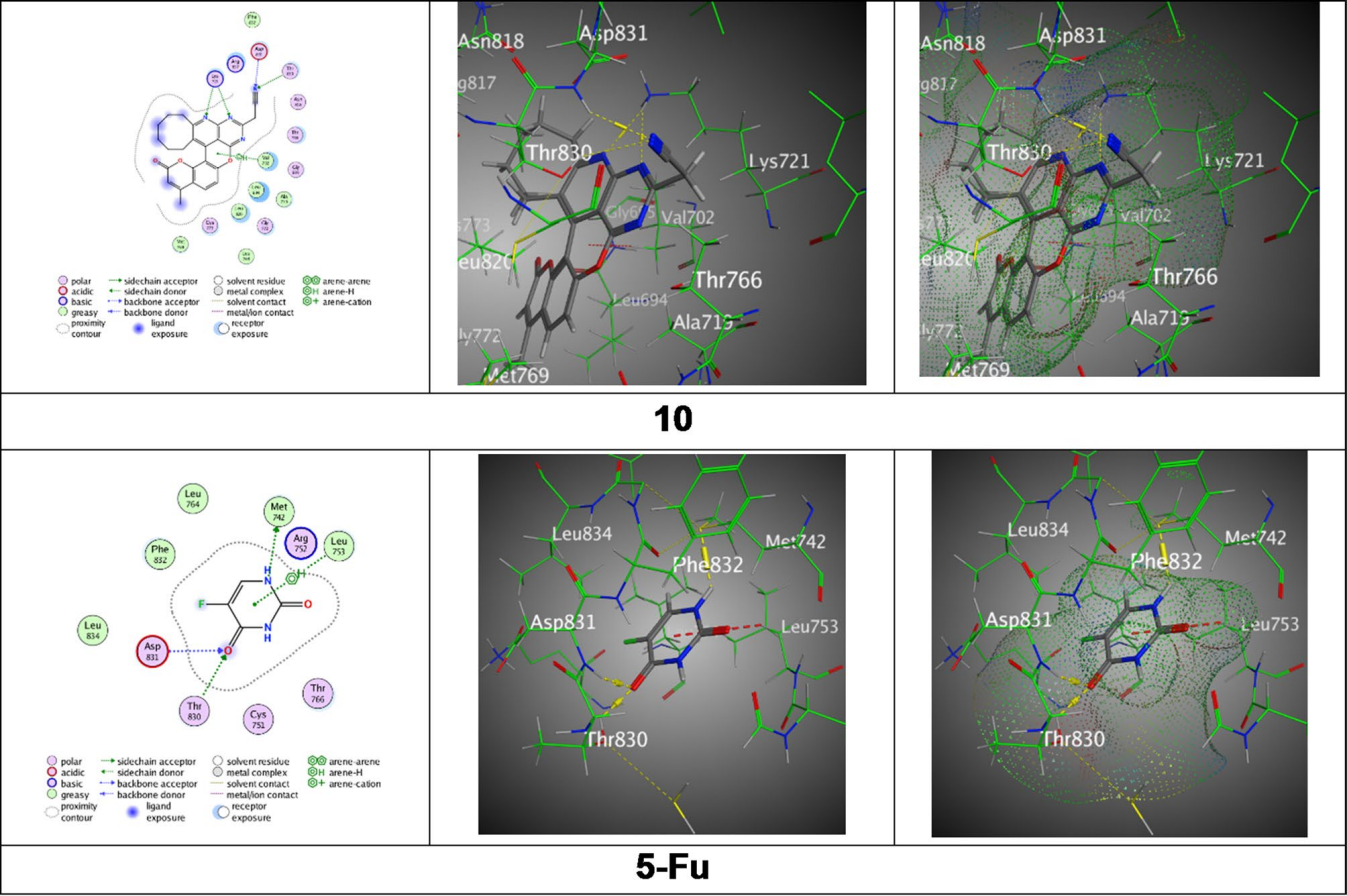
Binding images between ligands with PDB: **4HJO**.

The chromenone derivatives (**7–10**) were docked by captivating a selected PDBD: 4HJO protein, epidermal growth factor receptor (EGFR) using the M.O.E program and their results were documented (Table 2 and Fig. 6). Derivative **7** exhibited a binding energy of S = -7.5238 kcal/mol with RMSD = 1.5480. The bindings elaborated the N4 of the pyridine ring, N9 of the pyrimidine ring, and N29 of the nitrile group forming hydrogen bonds with Lys721 and Asp831, and a Pi-H interaction of the pyran-2-one ring with Leu 694, at distances ranging from 2.94 to 4.33 Å. Though derivative **8** Showed a binding energy of S= -7.4999 kcal/mol through RMSD = 1.4130. The bindings included hydrogen bonds between N3 and N8 of the pyridine and pyrimidine rings with Lys 721, and N30 of the nitrile group with Asp 831, with intermolecular distances ranging between 2.95 and 3.69 Å. Meanwhile, derivative **9** disclosed a binding energy of S= -7.1885 kcal/mol over RMSD = 1.4597. It engaged in Pi-H interaction of the pyrimidine ring with Phe699 and a Pi-cation interaction of the pyran-2-one ring with Lys721, with distances of 3.61 and 4.45 Å respectively. Moreover, derivative **10** achieved the lowest binding energy of S= -7.8247 kcal/mol through RMSD = 1.1874. It demonstrated multiple H-bond interactions with Lys721, Thr830, and Asp831 *via* N1 and N6 of the pyridine and pyrimidine rings and N32 of the nitrile group, along with a Pi-H interaction with Val 02, at distances from 2.31 to 3.99 Å. Furthermore, reference (5-Fu) Offered binding energy S= -4.4448 kcal/mol and an RMSD = 1.3762. The bindings included H-bonding of N1 of the pyrimidine ring with Met742, O6 of the amide group with Thr830 and Asp831, and a Pi-H interaction with Leu 53, through bond distances from 2.86 to 4.72 Å.

**Table 2:** Docking results of the synthesized derivatives.

Derivative	S (Kcal/mol)	RMSD	Ligand bindings with the amino-acid residues	Types of Interactions	Distance (Å)
**7**	-7.5238	1.5480	N 4 of pyridine ring with Lys 721 N 9 of pyrimidine ring with Lys 721 N 29 of nitrile group with Asp 831 Pyran-2-one ring with Leu 694	H-acceptor H-acceptor H-acceptor Pi-H	2.94 3.69 3.38 4.33
**8**	-7.4999	1.4130	N 3 of pyridine ring with Lys 721 N 8 of pyrimidine ring with Lys 721 N 30 of nitrile group with Asp 831	H-acceptor H-acceptor H-acceptor	2.95 3.69 3.28
**9**	-7.1885	1.4597	pyrimidine ring with Phe 699 Pyran-2-one ring with Lys 721	Pi-H Pi- cation	3.61 4.45
**10**	-7.8247	1.1874	N 1 of pyridine ring with Lys 721 N 6 of pyrimidine ring with Lys 72 N 32 of nitrile group with Thr 830 N 32 of nitrile group with Asp 831 Pyrane ring with Val 702	H-acceptor H-acceptor H-acceptor H-acceptor Pi-H	3.03 3.63 3.33 2.31 3.99
**5-Fu**	-4.4448	1.3762	N 1 of pyrimidine ring with Met 742 O 6 of amide group with Thr 830 O 6 of amide group with Asp 831 1 Pyrimidine ring with Leu 753	H-donor H-acceptor H-acceptor Pi-H	3.35 2.86 3.17 4.72

## Experiment

### Synthesis of 7-hydroxy-4-methyl-2-oxo-*2 H*-chromene-8-carbaldehyde (2)

It was prepared according to previously reported work^47^. Yield, 80%; m.p. 180–182 °C [Lit. 174–176 [32b]]. ^1^H-NMR (Fig. S1).

### Synthesis of 8-imino-4-methyl-2-oxo-*2 H*,*8 H*-pyrano[2,3-*f*]chromene-9-carbonitrile (3)

After two hours of refluxing a mixture of malononitrile (1.98 g, 0.03 mol), compound **2** (0.61 g, 0.03 mol), and ammonium acetate (3.85 g, 0.05 mol) in ethanol (25 mL), a yellowish product was obtained. Compound **3** was obtained by filtering the crystals, washing them in hot ethanol, and then recrystallizing them from methanol/benzene.

Yield, 72%; m.p. 232–234 °C [Lit. m.p. 229–231 [32b]; IR (KBr): *ν*_*max*_, cm^− 1^: 3250 (NH), 2220 (CN), 1733 (CO), 1620 (C = N), 1110 (C-O-C); ^1^H-NMR (DMSO-*d*_*6*_) *δ* ppm: 2.42 (s, 3 H, CH_3_), 6.18 (s, 1H, C_3_-H coumarin), 6.97 (d, 1H, *J* = 9.2 Hz, Ar-H), 7.59 (d, 1H, *J* = 9.0 Hz, Ar-H), 8.71 (s, 1H, C_10_-H pyrane), 9.36 (s, 1H, NH) (Fig. S2); ^13^C-NMR (DMSO-*d*_*6*_) *δ* ppm: 19.8, 97.0, 108.0, 110.5, 112.9 (2 C), 115.1, 118.2, 125.2, 146.1, 152.4, 154.1, 160.0, 160.1 (Figure S3). MS (m/z, %): 252 (M^+^, 60). Anal. Calced for C_14_H_8_N_2_O_3_ (252.23): C, 66.67; H, 3.20; N, 11.11%. Found: C, 66.59; H, 2.99; N, 11.07%.

### Synthesis of 2-amino-5-(cyanomethyl)-10-methyl-12-oxo-*12 H*-7,13-dioxa-3,4,6-triazabenzo[*no*]tetraphene-1-carbonitrile (5)

A mixture of malononitrile (4.96 g, 0.06 mol), compound **2** (0.61 g, 0.03 mol), and ammonium acetate (3.85 g, 0.05 mol) in ethanol (25 mL) was treated in similar manner as that described above for the synthesis of **(3)** to yield compound **5**.

Yield, 75%; m.p. 263–265 °C; IR (KBr): *ν*_*max*_, cm^− 1^: 3410 (NH_2_), 2219 (CN), 1738 (CO), 1615 (C = N), 1600 (C = C), 1115 (C-O-C); ^1^H-NMR (DMSO-*d*_*6*_) *δ* ppm: 2.43 (s, 3 H, CH_3_), 4.10 (s, 2 H, CH_2_), 6.30 (s, 1H, C_3_-H coumarin), 6.92 (s, 2 H, NH_2_), 7.21 (d, 1H, Ar-H), 7.93 (d, 1H, Ar-H). MS (m/z, %): 382 (M^+^, 66). Anal. Calced for C_20_H_10_N_6_O_3_ (382.34): C, 62.83; H, 2.64; N, 21.98%. Found: C, 62.77; H, 2.59; N, 21.87%.

### The reaction of compound 2 with malononitrile and acetophenone

(A) Reaction time of 2 h:

After two hours of refluxing a mixture of malononitrile (2.98 g, 0.03 mol), compound 2 (0.61 g, 0.03 mol), acetophenone (3.6 g, 0.03 mol), and ammonium acetate (3.85 g, 0.05 mol) in ethanol (25 mL), dark crystals were formed. After cooling the mixture to room temperature, the crystals were separated by filtration and refined using column chromatography with silica gel 100/160 and benzene: ethyl acetate (1:1) as the eluent. This yielded 16% of compound **4**, 12% of compound **5**, and twenty-five% of compound **6**.

### 2-(11-Imino-4-methyl-2-oxo-*2 H*,*11 H*-pyrano[2’,3’:5,6]chromeno[2,3-*d*]pyrimidin-9-yl) acetonitrile (4)

Yield, 16%; m.p. 232–234 °C; IR (KBr): *ν*_*max*_, cm^− 1^: 3355 (NH), 2218 (CN), 1737 (CO), 1620 (C = N), 1595 (C = C), 1112 (C-O-C); ^1^H-NMR (DMSO-*d*_*6*_) *δ* ppm: 2.16 (s, 3 H, CH_3_), 3.15 (s, 2 H, CH_2_), 6.05 (s, 1H, C_3_-H coumarin), 6.84 (d, 1H, Ar-H), 7.45 (d, 1H, Ar-H), 7.81 (s, 1H, C_10_-H pyrane), 11.90 (s, 1H, NH) (Fig. S4); ^13^C-NMR (DMSO-*d*_*6*_) *δ* ppm: 19.9, 22.9, 108.5 (2 C), 113.3 (2 C), 115.9, 118.9, 122.0, 125.8, 146.8, 153.0, 154.8, 158.1, 161.3 (2 C), 171.8 (Fig. S5). MS (m/z, %): 318 (M^+^, 67). Anal. Calced for C_17_H_10_N_4_O_3_ (318.29): C, 64.15; H, 3.17; N, 17.60%. Found: C, 64.10; H, 3.11; N, 17.52%.

### 2-Amino-5-(cyanomethyl)-10-methyl-12-oxo-*12 H*-7,13-dioxa-3,4,6-triazabenzo[*no*] tetraphene-1-carbonitrile (5)

Yield, 12%; m.p. 263–265 °C; IR (KBr): *ν*_*max*_, cm^− 1^: 3410 (NH_2_), 2219 (CN), 1738 (CO), 1615 (C = N), 1600 (C = C), 1115 (C-O-C); ^1^H-NMR (DMSO-*d*_*6*_) *δ* ppm: 2.43 (s, 3 H, CH_3_), 4.10 (s, 2 H, CH_2_), 6.30 (s, 1H, C_3_-H coumarin), 6.92 (s, 2 H, NH_2_), 7.21 (d, 1H, Ar-H), 7.93 (d, 1H, Ar-H) (Fig. S6). MS (m/z, %): 382 (M^+^, 66). Anal. Calced for C_20_H_10_N_6_O_3_ (382.34): C, 62.83; H, 2.64; N, 21.98%. Found: C, 62.77; H, 2.59; N, 21.87%.

### 2-(10-Methyl-12-oxo-2-phenyl-*12 H*-7,13-dioxa-3,4,6-triazabenzo[no]tetraphene-5-yl) acetonitrile (6)

Yield, 25%; m.p. 277–279 °C; IR (KBr): *ν*_*max*_, cm^− 1^: 2218 (CN), 1739 (CO), 1610 (C = N), 1599 (C = C), 1113 (C-O-C); ^1^H-NMR (DMSO-*d*_*6*_) *δ* ppm: 2.26 (s, 3 H, CH_3_), 3.88 (s, 2 H, CH_2_), 6.18 (s, 1H, C_3_-H coumarin), 6.99 (d, 1H, Ar-H), 7.22–7.41 (m, 4 H, Ar-H), 7.80 (s, 1H, C_10_-H pyrane), 8.40 (d, 2 H, Ar-H) (Fig. S7). MS (m/z, %): 418 (M^+^, 59). Anal. Calced for C_25_H_14_N_4_O_3_ (418.41): C, 71.77; H, 3.37; N, 13.39%. Found: C, 71.71; H, 3.30; N, 13.35%.

(B) Reaction time of 10 min:

A mixture of malononitrile (2.98 g, 0.03 mol), compound **2** (0.61 g, 0.03 mol), acetophenone (3.6 g, 0.03 mol), and ammonium acetate (3.85 g, 0.05 mol) in ethanol (25 mL) was refluxed for 10 min. and then worked up as described above in A to give (15%) of compound **(3)**, (10%) of compound **(6)**, and (7%) of compound **(4)**.

### Formation of compound 5 from compound 4

After two hours of refluxing a mixture of malononitrile (1.98 g, 0.03 mol), compound **4** (0.30 g, 0.03 mol), and ammonium acetate (3.85 g, 0.05 mol) in ethanol (25 mL), a yellowish product was obtained. Compound **5** was obtained by filtering the crystals, washing them in hot ethanol, and then recrystallizing them from methanol/benzene.

### The reaction of (3) with malononitrile and Cyclic ketones

#### General procedure

After two hours of refluxing a mixture of (3) (0.756 g, 0.003 mol), malononitrile (0.198 g, 0.003 mol), suitable ketone (0.003 mol), and ammonium acetate (0.385 g, 0.005 mol) in ethanol (25 mL), yellow crystals were obtained. Compounds **7–10** were produced by filtering the crystals and recrystallizing them from methanol.

### 2-(14-Methyl-12-oxo-9,10-dihydro-*8 H*,*12 H*-3,11-dioxa-4,6,7-triazaindeno[6,5,4-*no*] tetraphen-5-yl)acetonitrile (7)

Yield, 77%; m.p. 244–246 °C; IR (KBr): ν_max_, cm^− 1^: 2220 (CN), 1738 (CO), 1612 (C = N), 1596 (C = C), 1109 (C-O-C); ^1^H-NMR (DMSO-*d*_*6*_) *δ* ppm: 2.11–2.30 (m, 2 H, CH_2_), 2.32 (s, 3 H, CH_3_), 3.00 (t, 2 H, CH_2_), 3.11 (t, 2 H, CH_2_), 3.79 (s, 2 H, CH_2_), 6.25 (s, 1H, C_3_-H coumarin), 7.15 (d, 1H, Ar-H), 7.70 (d, 1H, Ar-H) (Fig. S8). MS (m/z, %): 382 (M^+^, 55). Anal. Calced for C_22_H_14_N_4_O_3_ (382.38): C, 69.10; H, 3.69; N, 14.65%. Found: C, 69.07; H, 3.56; N, 14.58%.

### 2-(15-Methyl-13-oxo-8,9,10,11-tetrahydro-*13 H*-3,12-dioxa-4,6,7-triazanaphtho[3,2,1-*no*] tetraphen-5-yl)acetonitrile (8)

Yield, 71%; m.p. 227–229 °C; IR (KBr): *ν*_*max*_, cm^− 1^: 2221 (CN), 1740 (CO), 1615 (C = N), 1600 (C = C), 1110 (C-O-C); ^13^C-NMR (DMSO-*d*_*6*_) *δ* ppm: 13.9, 22.7 (2 C), 24.9 (2 C), 29.1, 106.9, 112.0, 116.4 (2 C), 119.6, 126.1, 127.2, 136.0, 147.2, 150.0 (2 C), 151.8, 155.8, 157.9, 161.9, 163.5, 170.0 (Fig. S9). MS (m/z, %): 396 (M^+^, 68). Anal. Calced for C_23_H_16_N_4_O_3_ (396.41): C, 69.69; H, 4.07; N, 14.13%. Found: C, 69.58; H, 3.99; N, 14.10%.

### 2-(16-Methyl-14-oxo-9,10,11,12-tetrahydro-*8 H*,*14 H*-3,13-dioxa-4,6,7-triazacyclohepta[4,5]benzo[1,2,3-*no*]tetraphen-5-yl)acetonitrile (9)

Yield, 73%; m.p. 247–249 °C; IR (KBr): *ν*_*max*_, cm^− 1^: 2219 (CN), 1739 (CO), 1620 (C = N), 1601 (C = C), 1112 (C-O-C); ^13^C-NMR (DMSO-*d*_*6*_) *δ* ppm: 14.1, 23.5, 25.7, 28.0 (2 C), 33.5, 36.0, 106.0, 111.1, 115.8 (2 C), 118.7, 125.5, 126.2, 135.0, 146.2, 149.0 (2 C), 150.8, 154.9, 156.9, 161.0, 162.5, 169.2 (Fig. S10). MS (m/z, %): 410 (M^+^, 63). Anal. Calced for C_24_H_18_N_4_O_3_ (410.43): C, 70.23; H, 4.42; N, 13.65%. Found: C, 70.11; H, 4.37; N, 13.58%.

### 2-(17-Methyl-15-oxo-8,9,10,11,12,13-hexahydro-*15 H*-3,14-dioxa-4,6,7-triazacycloocta[4,5]benzo[1,2,3-*no*]tetraphen-5-yl)acetonitrile (10)

Yield, 70%; m.p. 252–254 °C; IR (KBr): *ν*_*max*_, cm^− 1^: 2220 (CN), 1738 (CO), 1621 (C = N), 1603 (C = C), 1111 (C-O-C); ^1^H-NMR (DMSO-*d*_*6*_) *δ* ppm: 0.95–1.20 (m, 4 H, 2CH_2_), 1.31–1.58 (m, 2 H, CH_2_), 1.70–1.95 (m, 2 H, CH_2_), 2.40 (s, 3 H, CH_3_), 2.78 (t, 2 H, CH_2_), 2.89 (t, 2 H, CH_2_), 3.81 (s, 2 H, CH_2_), 6.30 (s, 1H, C_3_-H coumarin), 7.14 (d, 1H, Ar-H), 7.92 (d, 1H, Ar-H) (Fig. S11). MS (m/z, %): 424 (M^+^, 61). Anal. Calced for C_25_H_20_N_4_O_3_ (424.46): C, 70.74; H, 4.75; N, 13.20%. Found: C, 70.69; H, 4.68; N, 13.11%.

### Reaction of (2) with Cyanoacetamide and ammonium acetate

#### 4-Methyl-2,8-dioxo-7,8-dihydro-*2 H*-pyrano[2,3-*f*]quinoline-9-carbonitrile (11)

A mixture of cyanoacetamide (2.52 g, 0.03 mol), compound **2** (0.61 g, 0.03 mol), and ammonium acetate (3.85 g, 0.03 mol) in ethanol (25 mL) was refluxed for 2 h and then worked up manner as described for the reaction of **(2)** with malononitrile and ammonium acetate to give compound **11**.

Yield, 78%; m.p. 247–249 °C; IR (KBr): *ν*_*max*_, cm^− 1^: 3335 (NH), 2218 (CN), 1741 (CO), 1675 (CO, amidic), 1615 (C = N), 1600 (C = C), 1110 (C-O-C); ^13^C-NMR (DMSO-*d*_*6*_) *δ* ppm: 13.3, 102.1, 112.0, 114.5 (3 C), 118.0, 120.3, 124.5, 136.2, 145.9, 152.0, 160.5, 168.0 (Fig. S12). MS (m/z, %): 252 (M^+^, 57). Anal. Calced for C_14_H_8_N_2_O_3_ (252.23): C, 66.67; H, 3.20; N, 11.11%. Found: C, 66.58; H, 3.15; N, 11.09%.

### The reaction of (2) with Cyanoacetamide and Cyclopentanone

(A) In the presence of ammonium acetate:

#### 8-Imino-4-methyl-2-oxo-*2 H*,*8 H*-pyrano[2,3-*f*]chromene-9-carboxamide (12)

A mixture of cyanoacetamide (2.52 g, 0.03 mol), compound **2** (0.61 g, 0.03 mol), cyclopentanone (2.52 g, 0.03 mol) and ammonium acetate (3.85 g, 0.05 mol) in ethanol (25 mL) was refluxed for 2 h and then worked up manner as described for the reaction of **(2)** with cyanoacetamide and ammonium acetate to give compound **12**.

Yield, 77%; m.p. 237–239 °C; IR (KBr): *ν*_*max*_, cm^− 1^: 3410 (NH_2_), 3250 (NH), 1738 (CO), 1680 (CO, amidic), 1610 (C = N), 1600 (C = C), 1111 (C-O-C); ^1^H-NMR (DMSO-*d*_*6*_) *δ* ppm: 2.44 (s, 3 H, CH_3_), 6.36 (s, 1H, C_3_-H coumarin), 6.90 (s, 2 H, NH_2_), 7.30 (d, 1H, Ar-H), 7.78 (d, 1H, Ar-H), 8.90 (s, 1H, C_10_-H pyrane), 9.54 (s, 1H, NH) (Fig. S13). MS (m/z, %): 270 (M^+^, 52). Anal. Calced for C_14_H_10_N_2_O_4_ (270.24): C, 62.22; H, 3.73; N, 10.37%. Found: C, 62.10; H, 3.68; N, 10.25%.

(B) In the presence of TEA:

A mixture of cyanoacetamide (2.52 g, 0.03 mol), compound **2** (0.61 g, 0.03 mol), cyclopentanone (2.52 g, 0.03 mol), and TEA (3.3 g, 0.05 mol) in ethanol (25 mL) was refluxed for 2 h and then worked up in the same manner as described for the reaction of **(2)** with cyanoacetamide, cyclopentanone, and ammonium acetate to give compound **14**.

Yield, 71%; m.p. 256–258 °C; IR (KBr): *ν*_*max*_, cm^− 1^: 3415 (NH_2_), 3155 (NH), 1725 (CO), 1680 (CO, amidic), 1600 (C = C), 1300 (C-O-C); ^13^C-NMR (DMSO-*d*_*6*_) *δ* ppm: 19.9 (2 C), 21.9, 30.2, 31.8, 82.8, 112.4 (2 C), 114.7 (2 C), 119.0, 122.1, 129.9, 152.1, 154.0 (2 C), 157.1, 158.6, 160.4. MS (m/z, %): 336 (M^+^, 50). Anal. Calced for C_19_H_16_N_2_O_4_ (336.35): C, 67.85; H, 4.80; N, 8.33%. Found: C, 67.77; H, 4.69; N, 8.21%.

### Preparation of authentic samples of (13) and (14)

A mixture of compound **(11)** (2.50 g, 0.01 mol), or **(12)** (2.70 g, 0.01 mol), cyclopentanone (0.83 g, 0.01 mol), and ammonium acetate (1.9 g, 0.25 mol) in ethanol (25 mL) was refluxed for 2 h during which yellow crystals appeared. The yellow crystals were obtained by filtration and recrystallized from dioxane to give 2.10 g (70%) of compound **13** and (82%) of compound **14**.

### 2-Amino-7-methyl-*10c*,11,12,13-tetrahydro-*3 H*-cyclopenta[5,6]pyrano[3,4-*c*]pyrano[2,3-*f*]quinoline-3,9(*4 H*)-dione (13)

Yield, 70%; m.p. 279–281 °C; IR (KBr): *ν*_*max*_, cm^− 1^: 3410 (NH_2_), 3157 (NH), 1730 (CO), 1685 (CO, amidic), 1605 (C = C), 1167 (C-O-C); ^1^H-NMR (DMSO-*d*_*6*_) *δ* ppm: 1.66–1.80 (m, 2 H, CH_2_), 2.19 (t, 4 H, 2CH_2_), 2.31 (s, 3 H, CH_3_), 3.84 (s, 1H, CH), 6.15 (s, 1H, C_3_-H coumarin), 6.68 (s, 2 H, NH_2_), 7.00 (d, 1H, Ar-H), 7.60 (d, 1H, Ar-H), 10.70 (s, 1H, NH) (Fig. S14). MS (m/z, %): 336 (M^+^, 53). Anal. Calced for C_19_H_16_N_2_O_4_ (336.35): C, 67.85; H, 4.80; N, 8.33%. Found: C, 67.79; H, 4.71; N, 8.25%.

### 3-Amino-7-methyl-*10c*,11,12,13-tetrahydro-*9 H*-cyclopenta[*b*]pyrano [2’,3’:5,6]chromeno [4,3-*d*]pyridine-2,9(*1 H*)-dione (14)

Yield, 82%; m.p. 256–258 °C; IR (KBr): *ν*_*max*_, cm^− 1^: 3415 (NH_2_), 3155 (NH), 1725 (CO), 1680 (CO, amidic), 1600 (C = C), 1300 (C-O-C); ^1^H-NMR (DMSO-*d*_*6*_) *δ* ppm: 1.91 (centered) (m, 2 H, CH_2_), 2.35 (t, 4 H, 2CH_2_), 2.44 (s, 3 H, CH_3_), 4.18 (s, 1H, CH), 6.32 (s, 1H, C_3_-H coumarin), 6.75 (s, 2 H, NH_2_), 6.90 (d, 1H, Ar-H), 7.56 (d, 1H, Ar-H), 12.40 (s, 1H, NH); ^13^C-NMR (DMSO-*d*_*6*_) *δ* ppm: 19.9 (2 C), 21.9, 30.2, 31.8, 82.8, 112.4 (2 C), 114.7 (2 C), 119.0, 122.1, 129.9, 152.1, 154.0 (2 C), 157.1, 158.6, 160.4 (Fig. S15). MS (m/z, %): 336 (M^+^, 50). Anal. Calced for C_19_H_16_N_2_O_4_ (336.35): C, 67.85; H, 4.80; N, 8.33%. Found: C, 67.77; H, 4.69; N, 8.21%.

### Reaction of compound 2, Cyanoacetamide and ammonium acetate with various ketones in acetic acid

A mixture of cyanoacetamide (2.52 g 0.03 mol), compound **2** (0.61 g, 0.03 mol), ammonium acetate (3.85 g, 0.05 mol), and 0.03 mol of either cyclopentanone, isopropyl methyl ketone, or acetophenone in acetic acid (25 mL) was refluxed for 2 h and the resultant yellow material recrystallized from dioxane to give (80%) of compound **15**.

### 4-Methyl-2,8-dioxo-*2 H*,*8 H*-pyrano[2,3-*f*]chromene-9-carbonitrile (15)

Yield, 80%; m.p. 277–279 °C [Lit. m.p. 272–274 [32b]; IR (KBr): *ν*_*max*_, cm^− 1^: 2220 (CN), 1737 (CO), 1605 (C = C), 1270 (C-O-C); ^1^H-NMR (DMSO-*d*_*6*_) *δ* ppm: 2.26 (s, 3 H, CH_3_), 6.31 (s, 1H, C_3_-H coumarin), 7.25 (d, 1H, *J* = 9.0 Hz, Ar-H), 7.71 (d, 1H, *J* = 9.0 Hz, Ar-H), 8.88 (s, 1H, C_10_-H pyrane) (Fig. S16). MS (m/z, %): 253 (M^+^, 59). Anal. Calced for C_14_H_7_NO_4_ (253.21): C, 66.41; H, 2.79; N, 5.53%. Found: C, 66.34; H, 2.72; N, 5.45%.

### Reaction of compound 2 and Ethyl cyano- acetate in the presence of ammonium acetate

A mixture of compound **2** (0.61 g, 0.03 mol), ammonium acetate (3.85 g, 0.05 mol), and ethyl cyanoacetate (3.45 g, 0.03 mol) in ethanol (25 mL) was refluxed for 2 h and the resulting yellow solid was recrystallized from dioxane to yield compound **16**.

Yield, 70%; m.p. 201–203 °C [Lit. m.p. 190–192 [32b]; IR (KBr): *ν*_*max*_, cm^− 1^: 3257 (NH), 1737 (2CO), 1620 (C = N), 1150 (C-O-C); ^13^C-NMR (DMSO-*d*_*6*_) *δ* ppm: 14.5, 19.2, 61.9, 108.1, 112.8 (2 C), 115.7, 118.6, 125.4, 129.0, 146.4, 152.6, 154.2, 161.0 (2 C), 165.9 (Fig. S17). MS (m/z, %): 299 (M^+^, 60). Anal. Calced for C_16_H_13_NO_5_ (299.28): C, 64.21; H, 4.38; N, 4.68%. Found: C, 64.13; H, 4.29; N, 4.60%.

### Reaction of compound 2, Ethyl cyanoacetate, and Cyclopentanone in the presence of ammonium acetate

A mixture of compound **2** (0.61 g, 0.03 mol), ammonium acetate (3.85 g, 0.05 mol), ethyl cyanoacetate (3.45 g, 0.03 mol), and cyclopentanone (2.46 g, 0.03 mol) in ethanol (25 mL) was refluxed for 2 h to yield 6compound **17**.

### 3-Imino-7-methyl-3,11,12,13-tetrahydro-*2 H*,*9 H*-cyclopenta[5,6]pyrano[3,4-*c*]pyrano [2,3-*f*]chromene-2,9-dione (17)

Yield, 76%; m.p. 281–283 °C; IR (KBr): *ν*_*max*_, cm^− 1^: 3133 (NH), 1723 (CO), 1606 (C = C), 1150 (C-O-C); ^13^C-NMR (DMSO-*d*_*6*_) *δ* ppm: 18.0, 20.0, 30.7, 35.4, 105.9, 108.8, 112.9 (2 C), 115.4, 118.6, 125.3, 140.3, 146.1, 152.6, 154.0, 160.6 (2 C), 162.3, 164.4 (Fig. S18). MS (m/z, %): 335 (M^+^, 52). Anal. Calced for C_19_H_13_NO_5_ (335.32): C, 68.06; H, 3.91; N, 4.18%. Found: C, 67.99; H, 3.82; N, 4.09%.

### Reaction of compound 2, ethyl cyanoacetate and ammonium acetate in ethanol

A mixture of compound **2** (0.61 g, 0.03 mol), ammonium acetate (3.85 g, 0.05 mol), and ethyl cyanoacetate (3.45 g, 0.03 mol) in ethanol (25 mL) was refluxed for 2 h to yield compound **18**.

### Ethyl 4-methyl-2,8-dioxo-*2 H*,*8 H*-pyrano[2,3-*f*]chromene-9-carboxylate (18)

Yield, 70%; m.p. 250–252 °C [Lit. m.p. 255–257 [32b]]; IR (KBr): *ν*_*max*_, cm^− 1^: 1735 (2CO), 1600 (C = C), 1165 (C-O-C); ^1^H-NMR (DMSO-*d*_*6*_) *δ* ppm: 1.45 (t, 3 H, *J* = 7.3 Hz, CH_3_), 2.30 (s, 3 H, CH_3_), 4.30 (q, 2 H, *J* = 7.2 Hz, CH_2_), 6.25 (s, 1H, C_3_-H coumarin), 7.19 (d, 1H, *J* = 9.0 Hz, Ar-H), 7.65 (d, 1H, *J* = 9 Hz, Ar-H), 8.80 (s, 1H, C_10_-H pyrane) (Fig. S19). MS (m/z, %): 300 (M^+^, 65). Anal. Calced for C_16_H_12_O_6_ (300.27): C, 64.00; H, 4.03%. Found: C, 63.92; H, 3.94%.

### Cytotoxic assay

It was carried out according to the previously reported work^48^.

### Molecular docking

The online version of M.O.E 2019 was utilized for molecular docking experiments. As a result, any molecule that inhibits the growth of EGFR-expressing malignancies might be regarded a significant target for anticancer therapy development. The protein EGFR with PDBD: 4HJO can be obtained from the Protein Data Bank at 10.2210/pdb4HJO/pdb. Meanwhile, heteroatoms and water are removed from the protein, energy is minimized, and ten poses are needed for effective docking^49^.

## Conclusion

7-Hydroxy-4-methyl-2-oxo-2 H-chromene-8-carbaldehyde **(2)** was used as a starting material to synthesize several pyridopyrimidine moieties linked to the coumarin ring by direct condensation of malononitrile with compound **2** in the presence of different ketones and ammonium acetate. The cytotoxicity study of the new compounds against HepG2, WI-38, VERO, and MCF-7 cell lines showed that compounds **8**, **9**, **10**, and **7** have the highest activity. Among the estimated derivatives from the docking study, derivative **10** recorded the highest binding affinity and the best docking score of -7.8247 kcal/mol, indicating its potential as a chief derivative for further development. The miscellaneous types of bindings identified, notably H-bonds and Pi-H interactions, highlight the significance of these derivatives’ structural properties in binding effectiveness.

## Electronic supplementary material

Below is the link to the electronic supplementary material.

Supplementary Material 1

## Data Availability

All data generated or analyzed during this study are included in this published article and its supplementary information files.
