# Supplementary material for: Design and synthesis of novel pyridopyrimidine moieties linked to coumarin ring of potentially active cytotoxic agents and their docking study
Source: Sci Rep. 2025 Jun 20;15:20155. doi: 10.1038/s41598-025-05325-1 (PMC12181260; doi:10.1038/s41598-025-05325-1)
Supplement: Supplementary file 1 — Supplementary Material 1 [file 41598_2025_5325_MOESM1_ESM.docx]

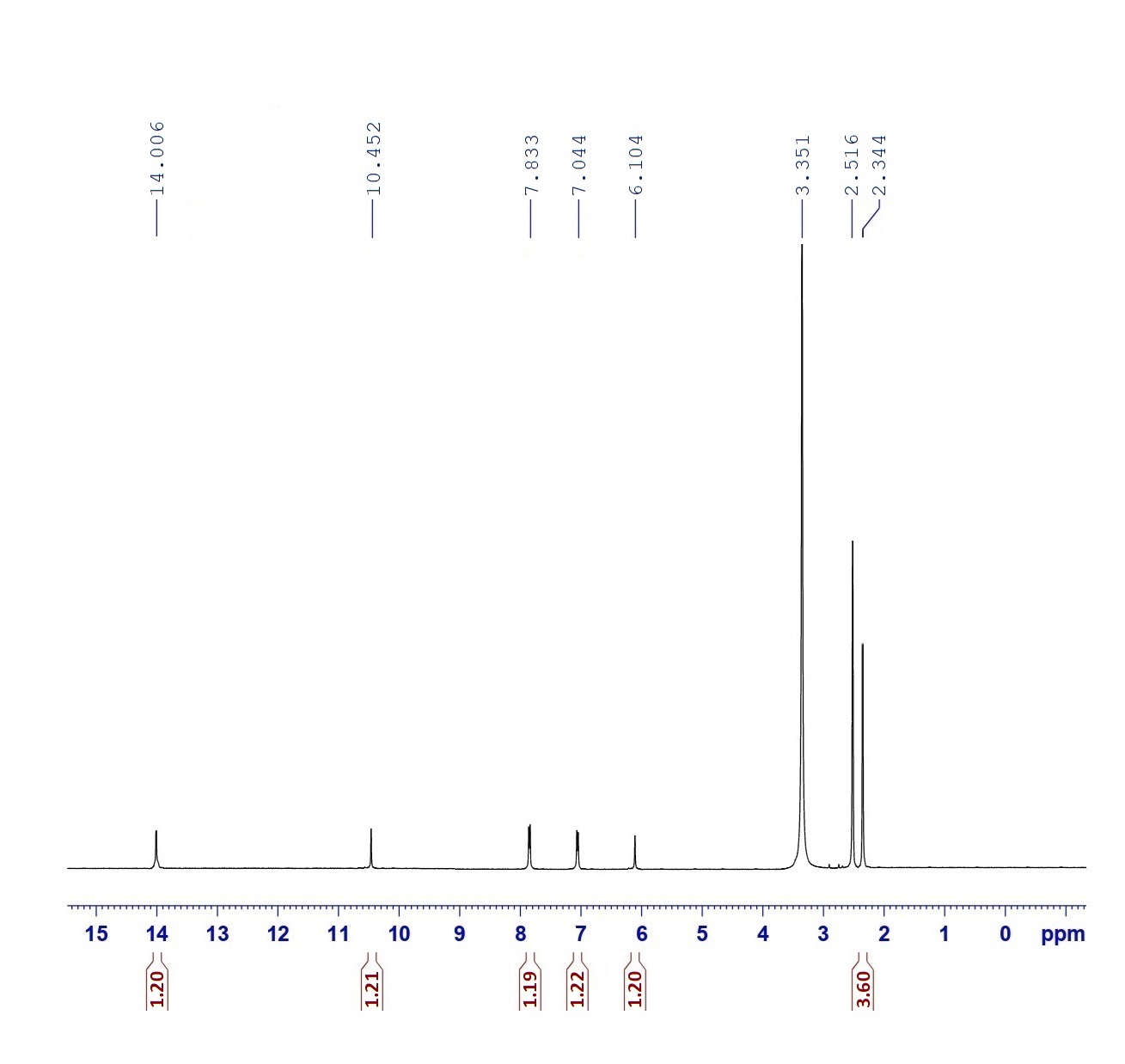


**Figure S1.** ^1^H-NMR of compound **2**


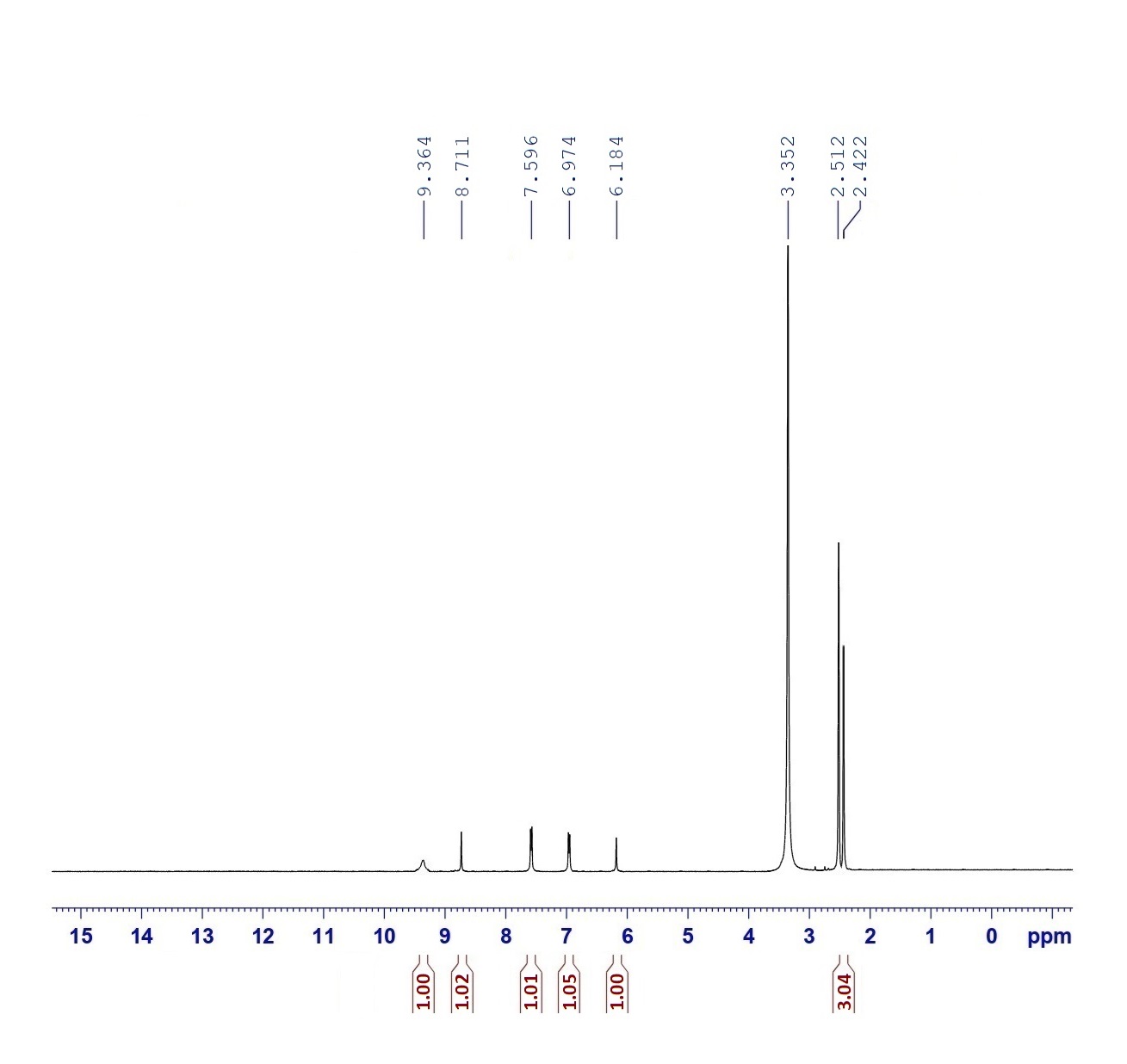


**Figure S2.** ^1^H-NMR of compound **3**

**
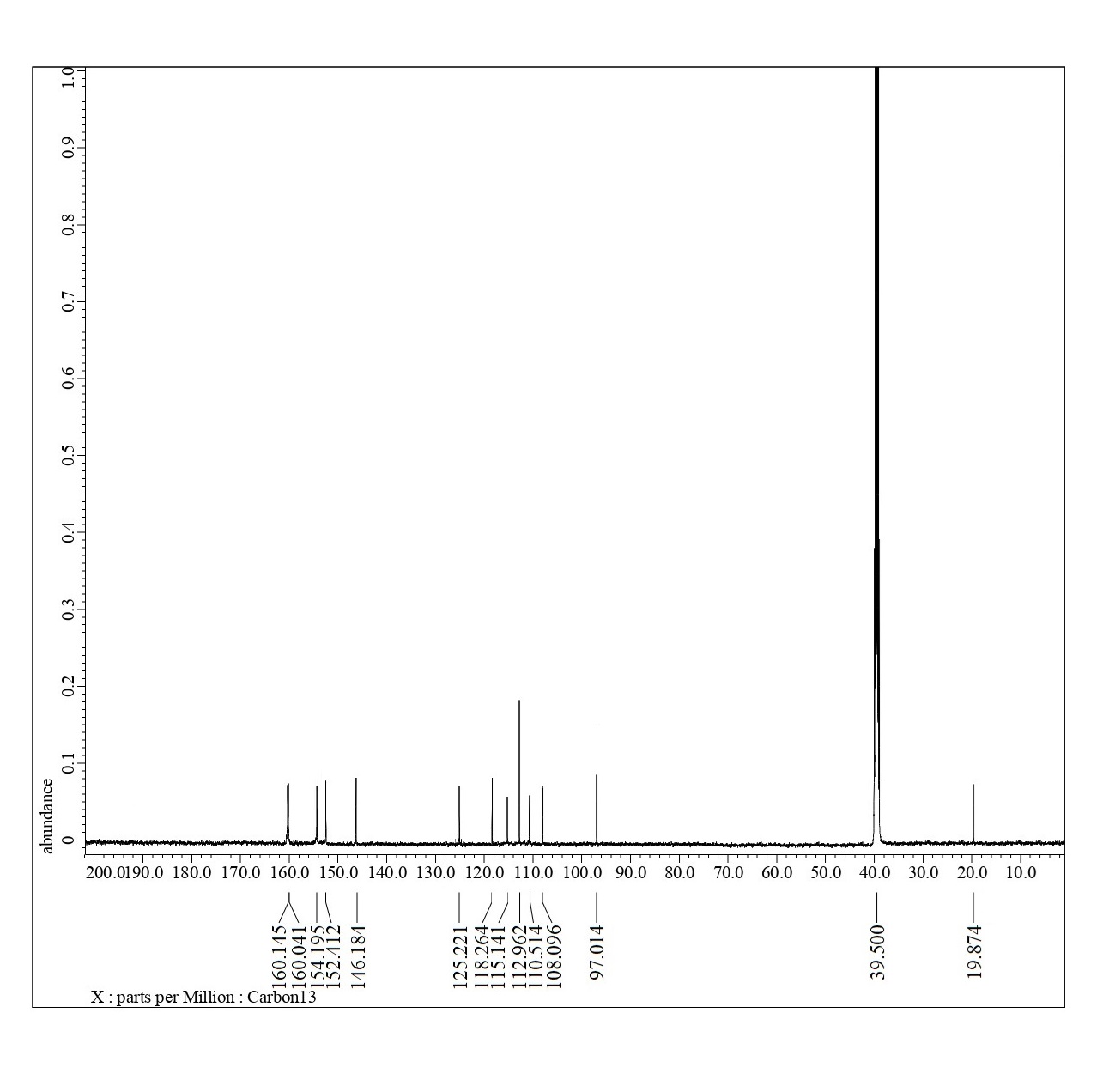
**

**Figure S3.** ^13^C-NMR of compound **3**


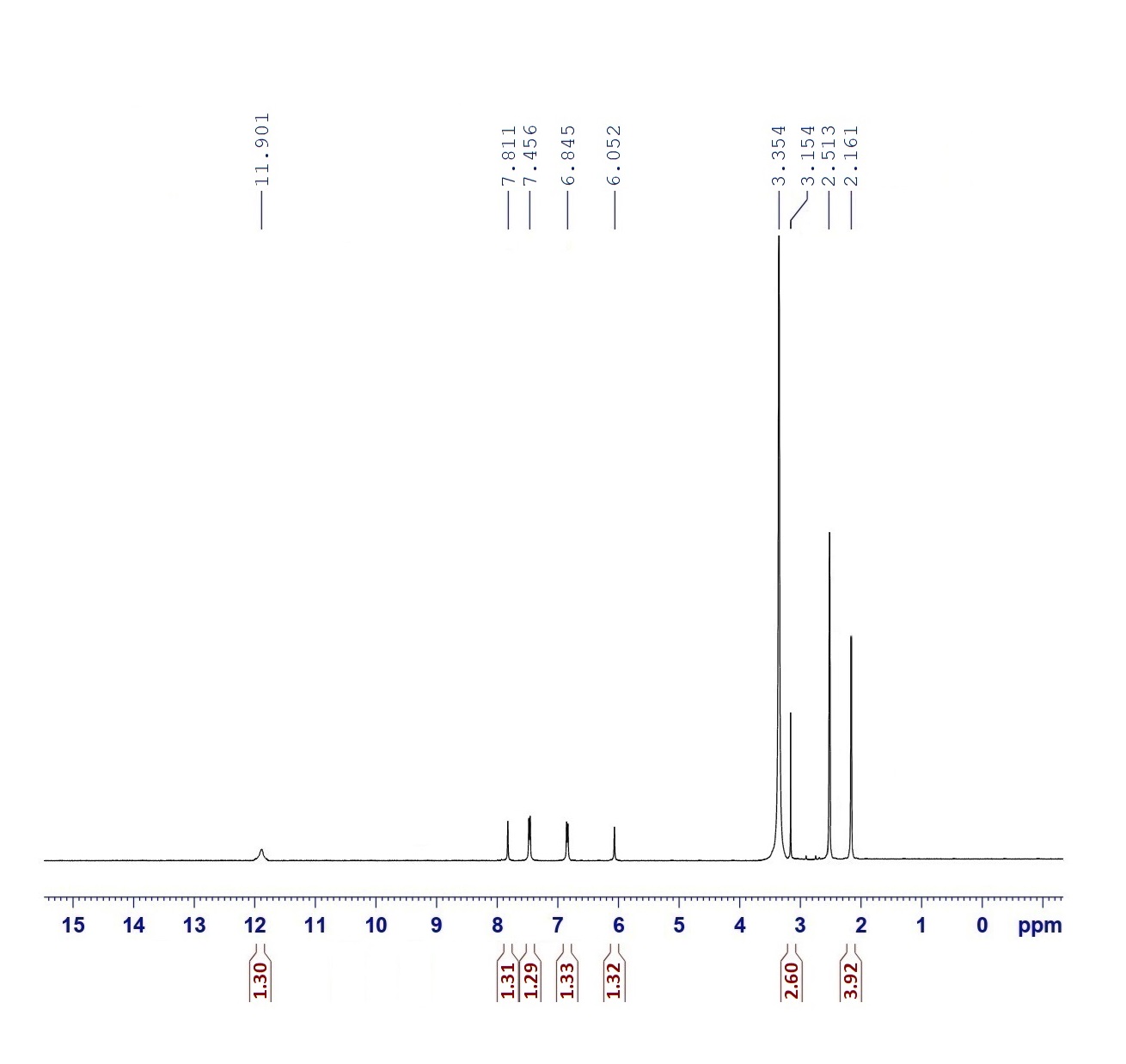


**Figure S4.** ^1^H-NMR of compound **4**

**
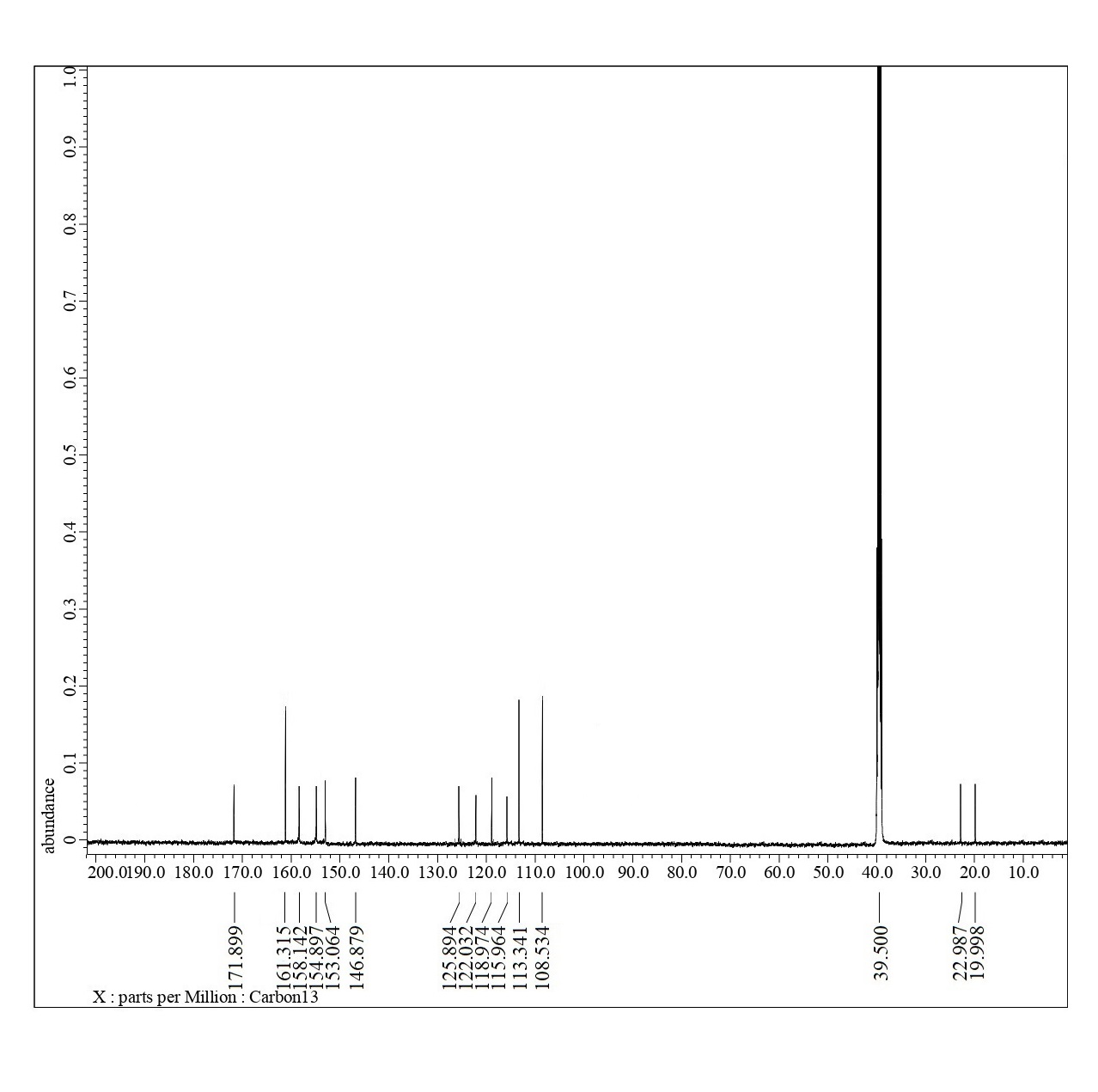
**

**Figure S5.** ^13^C-NMR of compound **4**


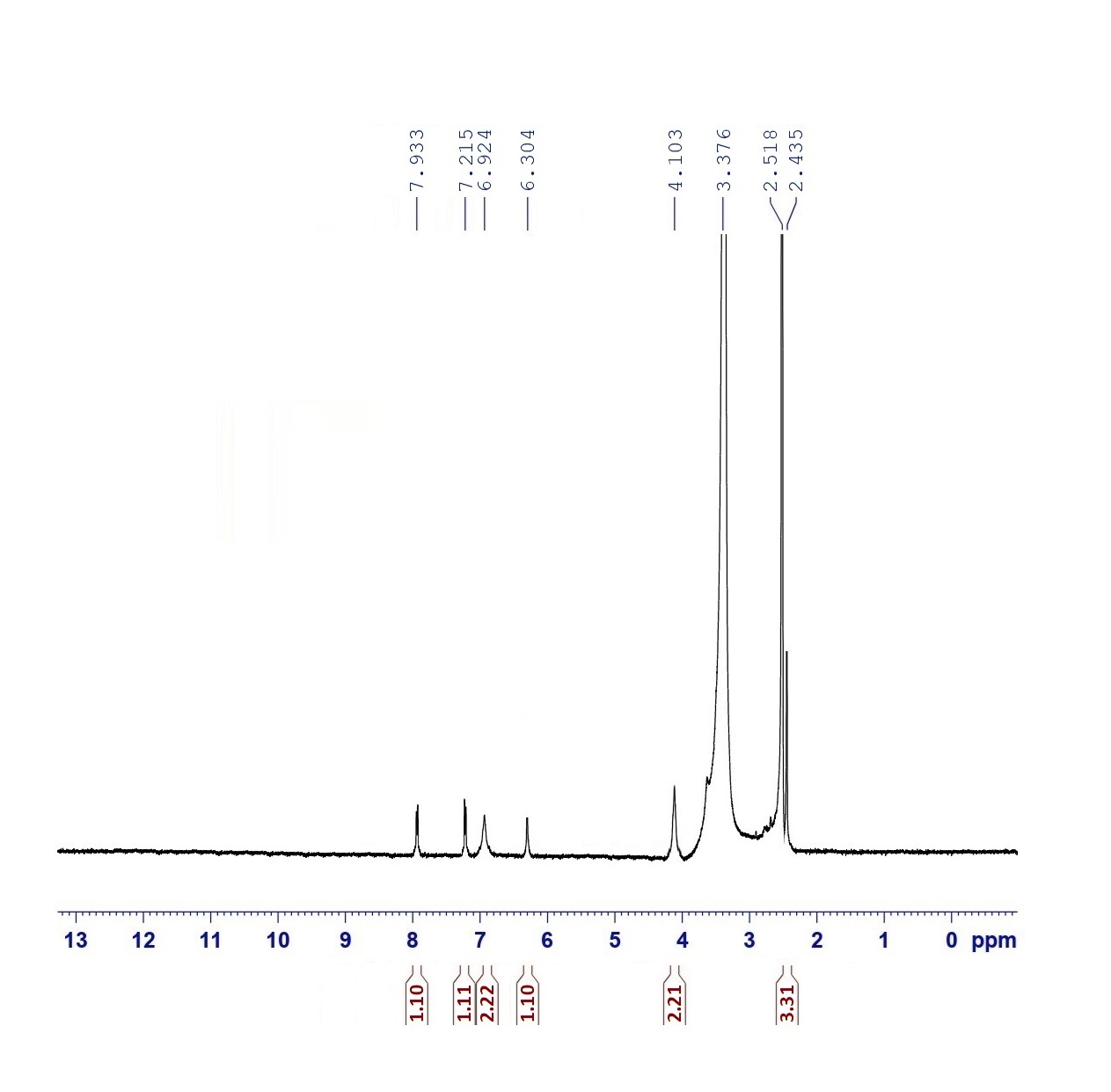


**Figure S6.** ^1^H-NMR of compound **5**


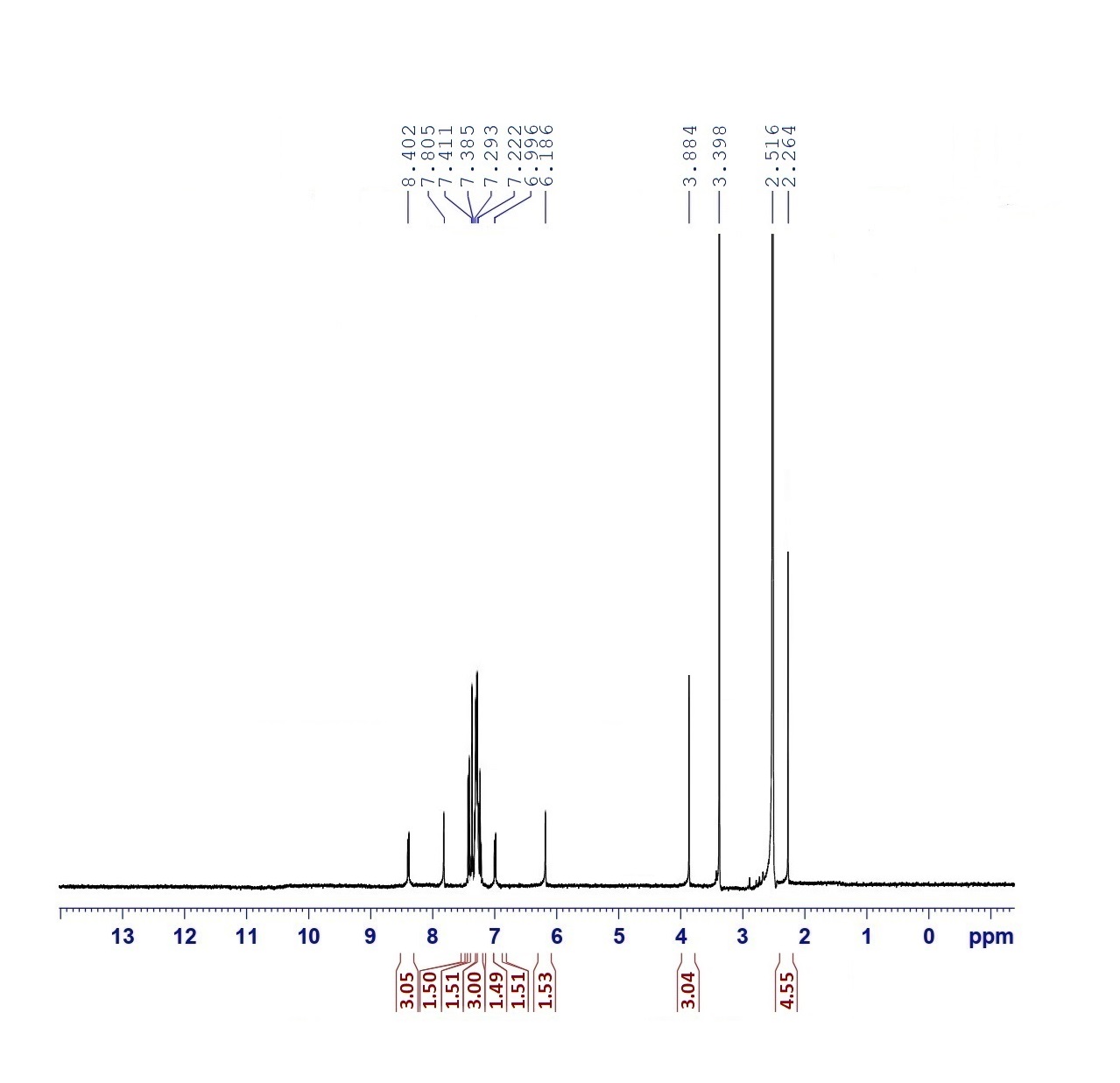


**Figure S7.** ^1^H-NMR of compound **6**


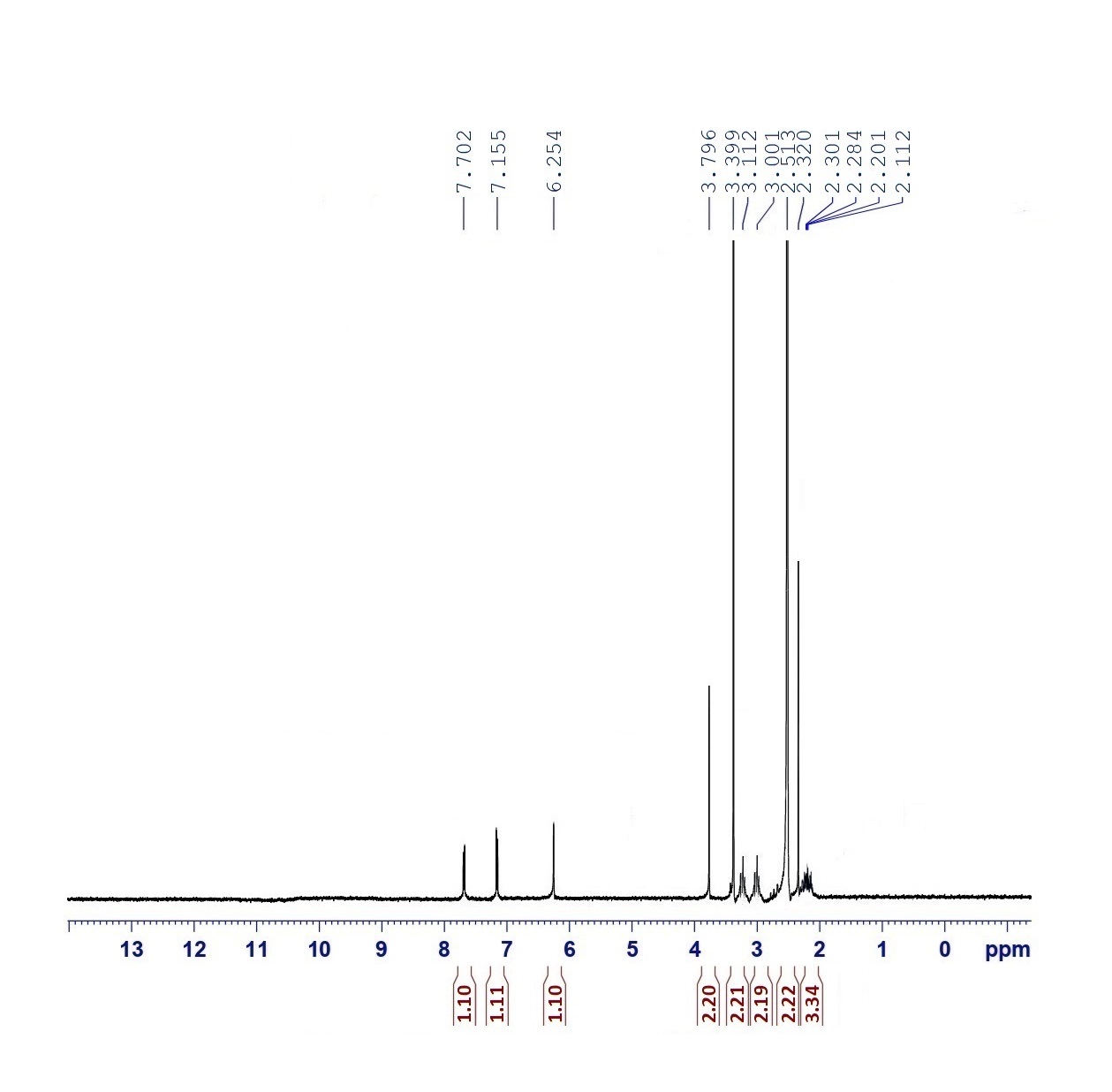


**Figure S8.** ^1^H-NMR of compound **7**

**
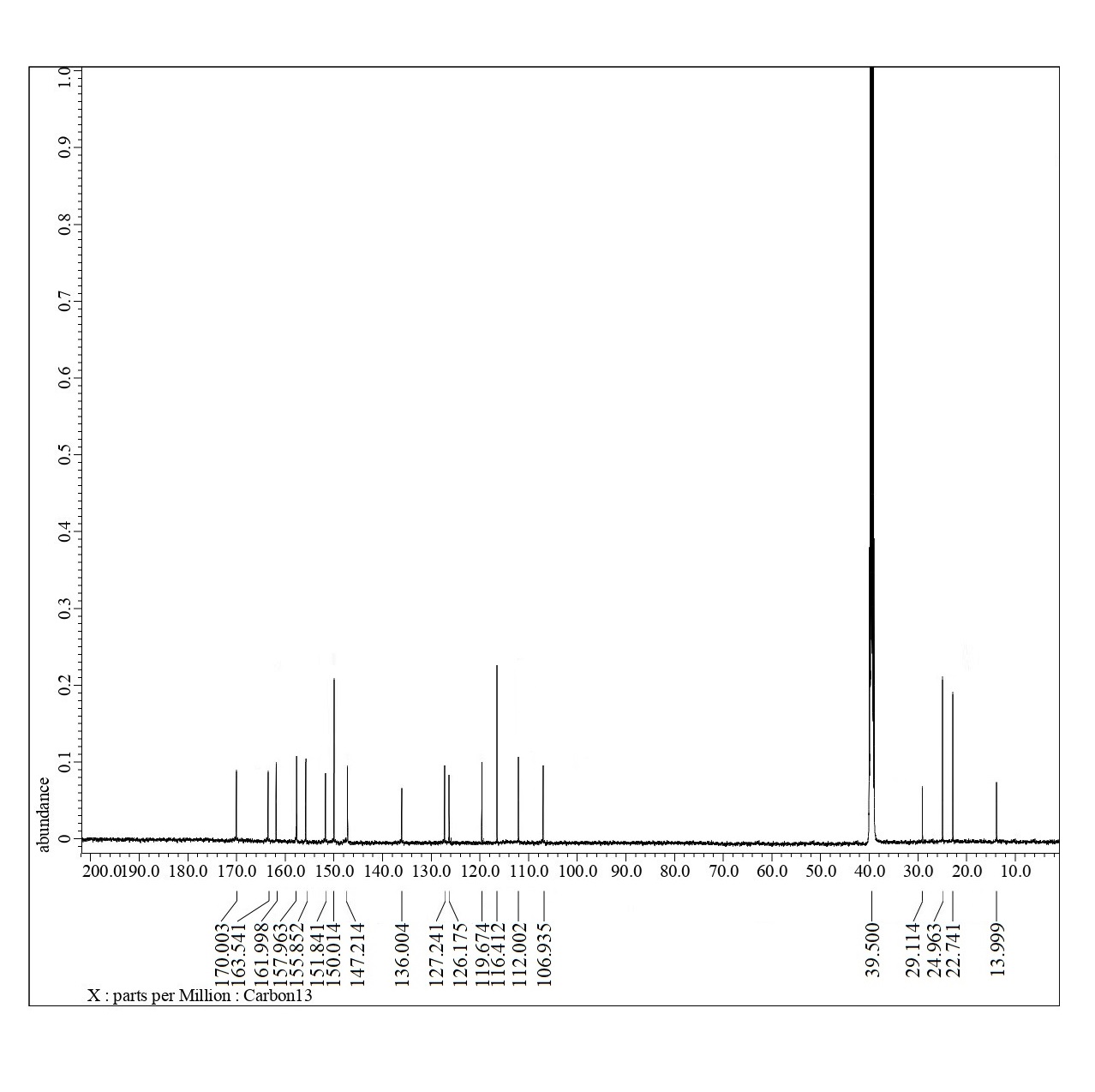
**

**Figure S9.** ^13^C-NMR of compound **8**


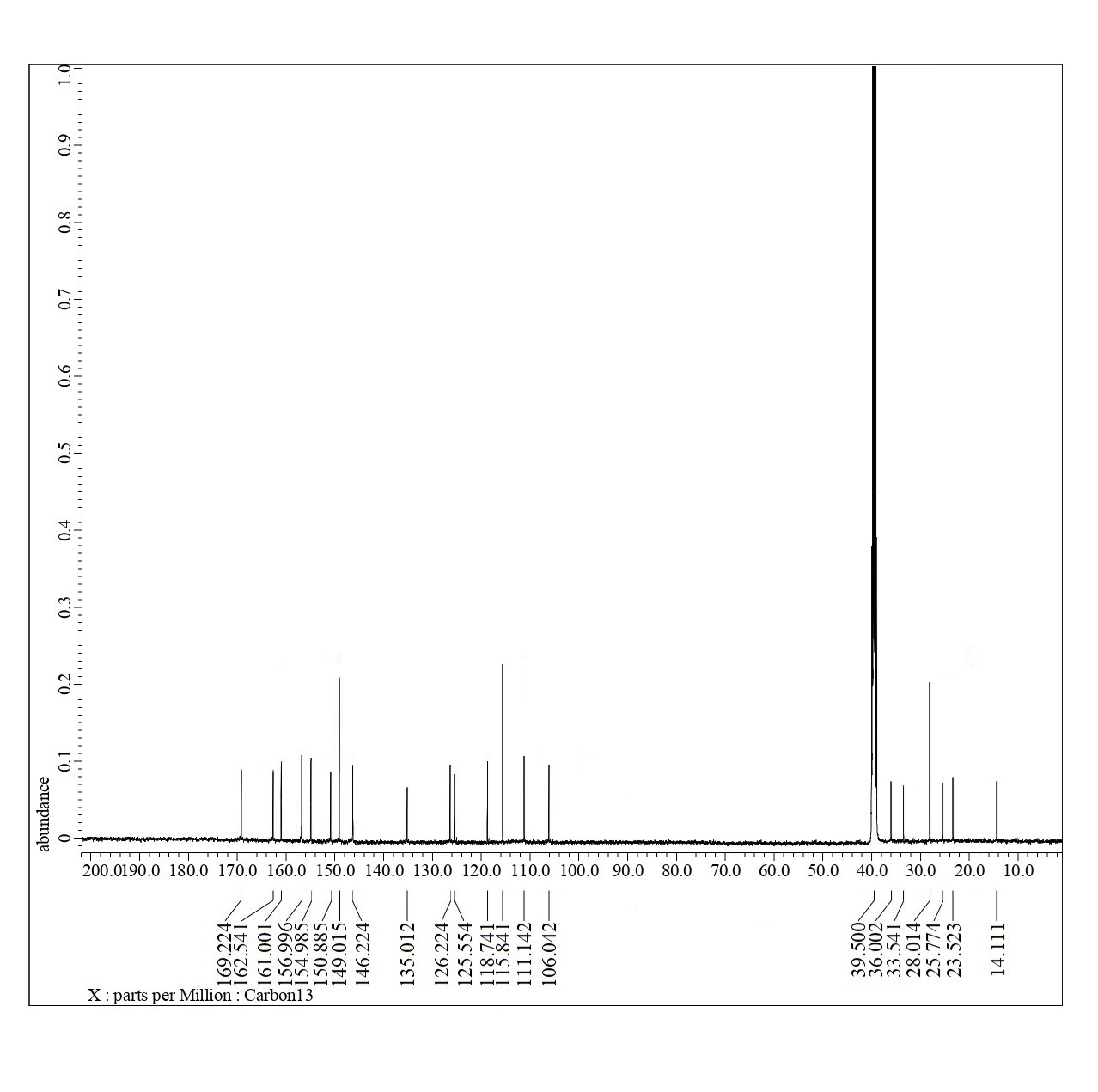


**Figure S10.** ^13^C-NMR of compound **9**


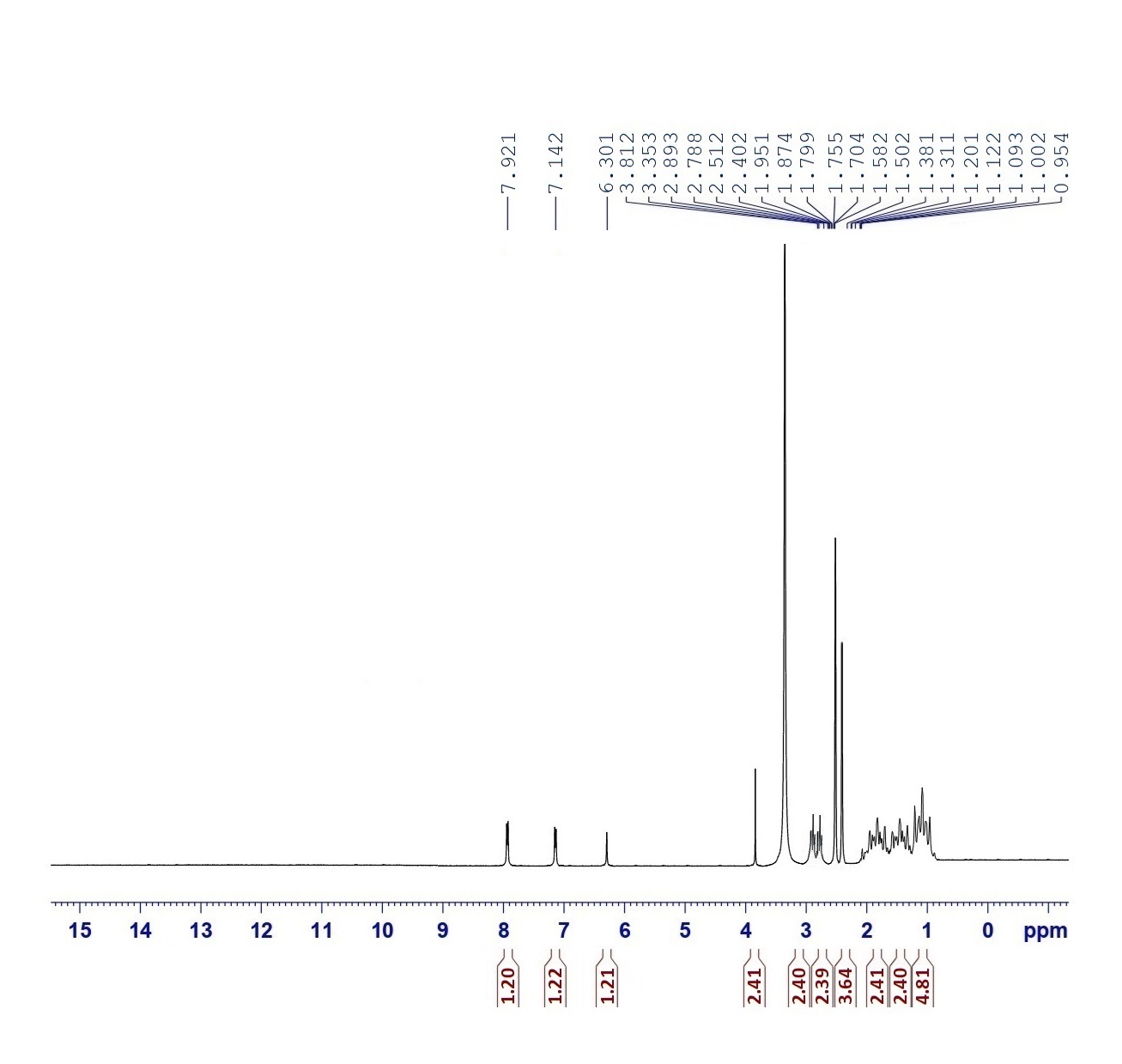


**Figure S11.** ^1^H-NMR of compound **10**

**
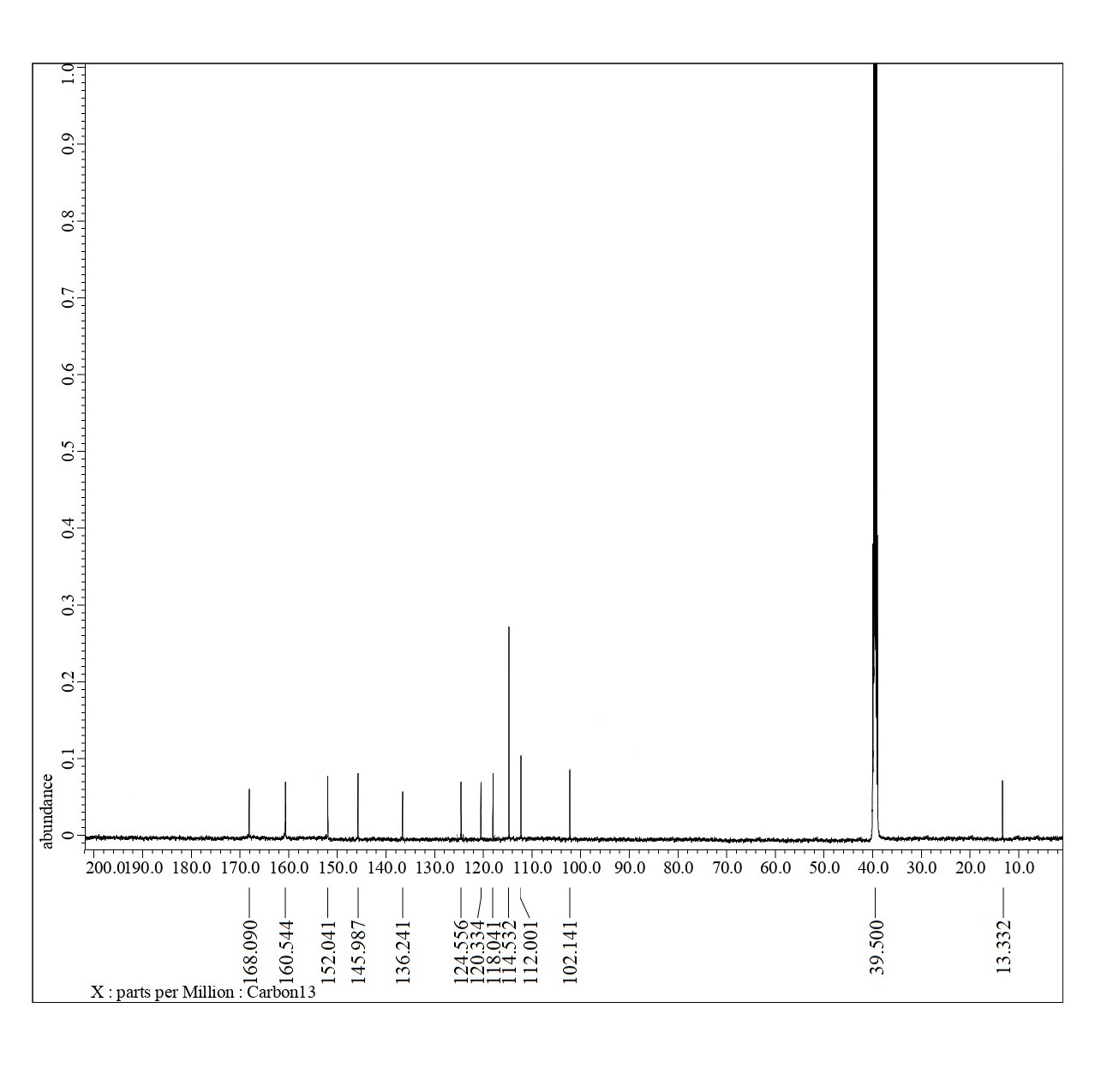
**

**Figure S12.** ^13^C-NMR of compound **11**


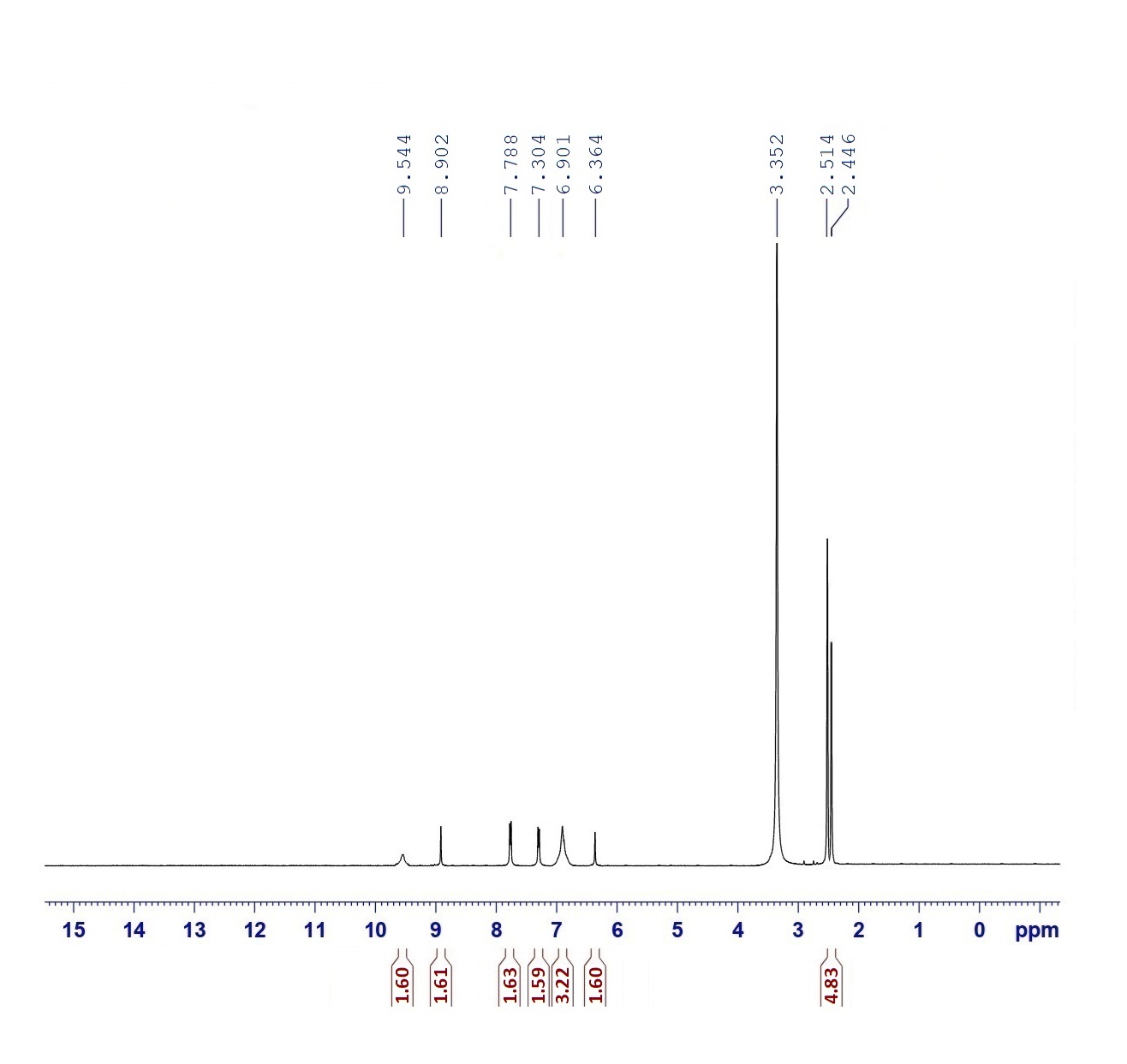


**Figure S13.** ^1^H-NMR of compound **12**


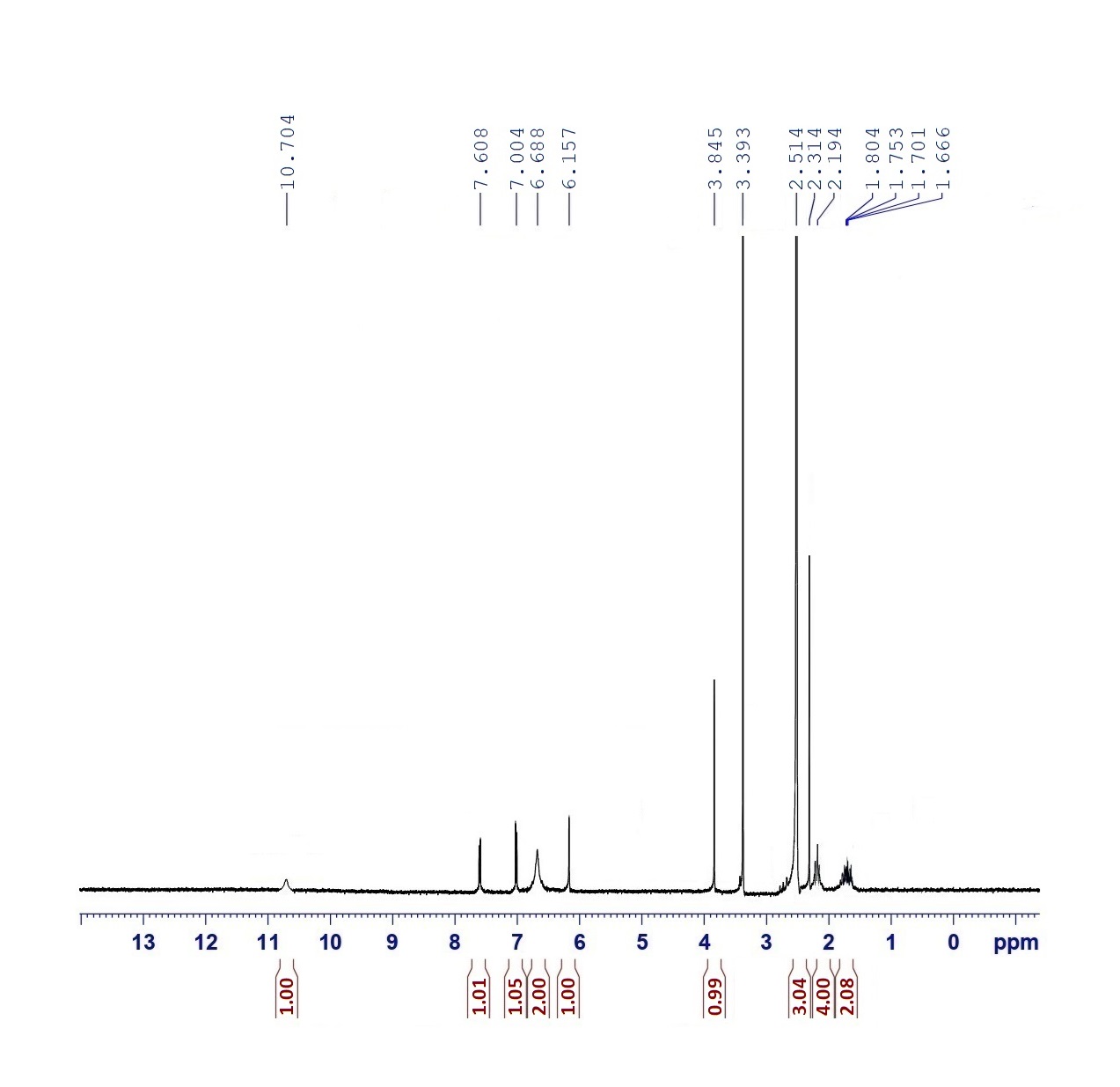


**Figure S14.** ^1^H-NMR of compound **13**

**
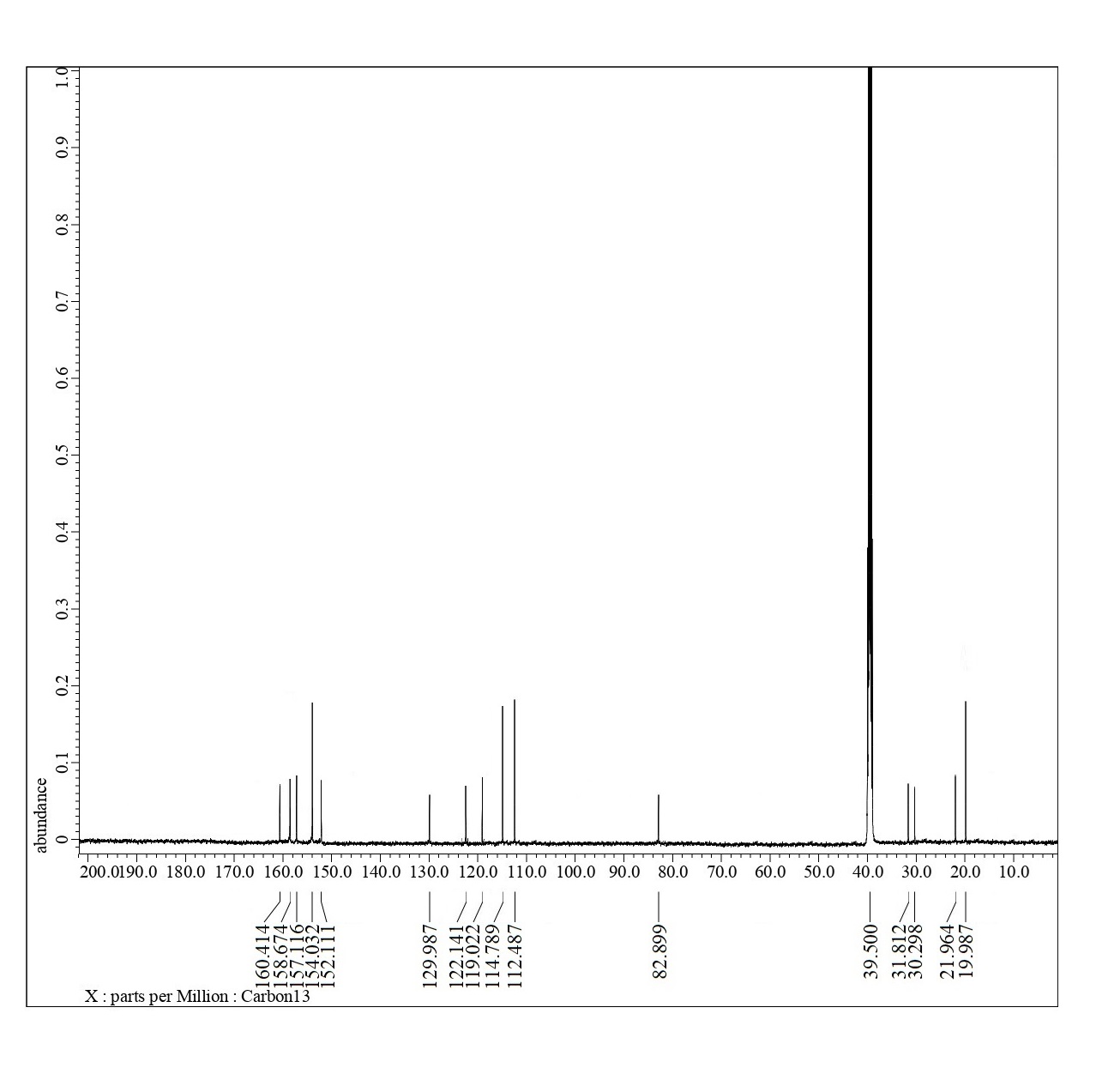
**

**Figure S15.** ^13^C-NMR of compound **14**


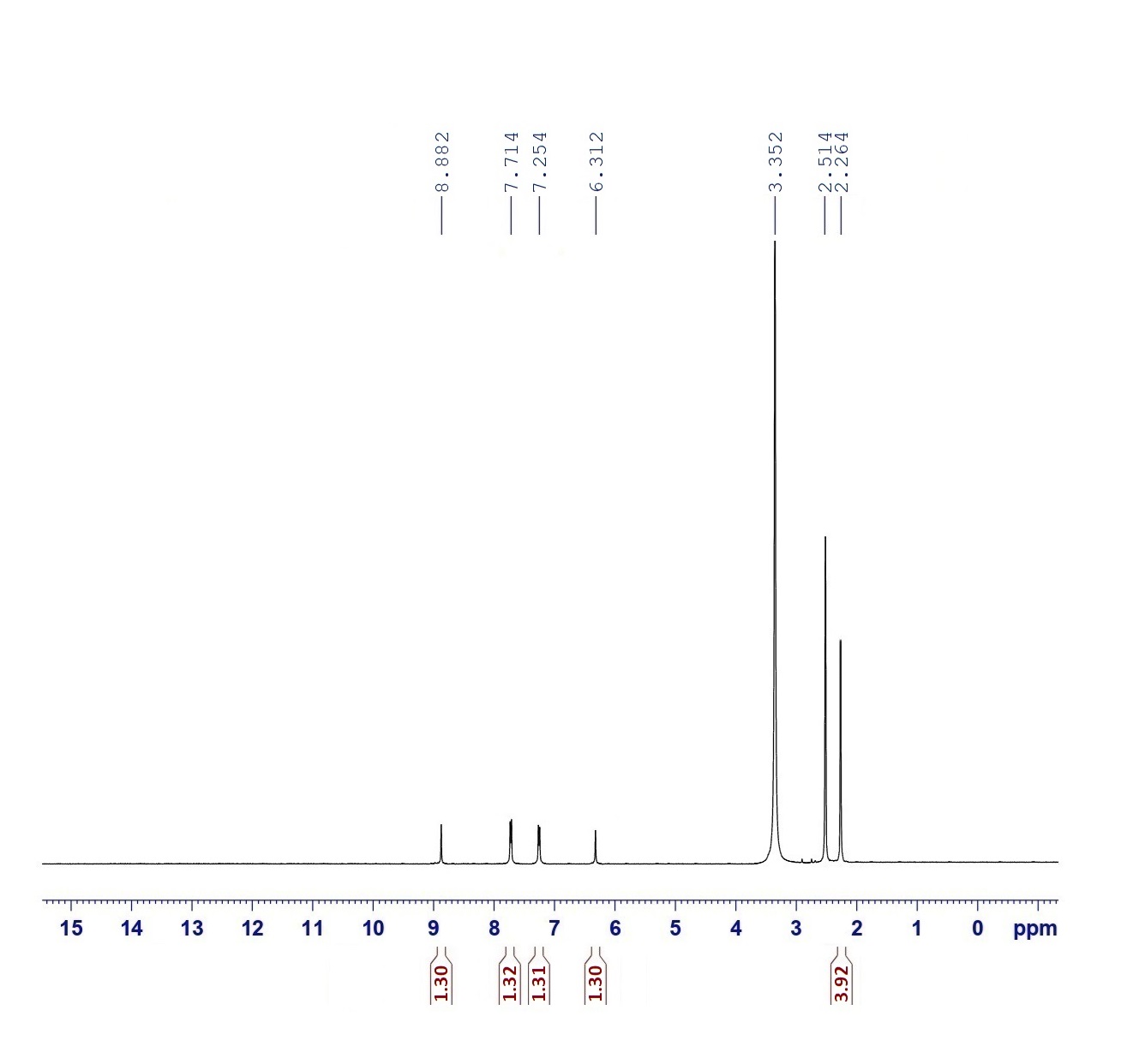


**Figure S16.** ^1^H-NMR of compound **15**

**
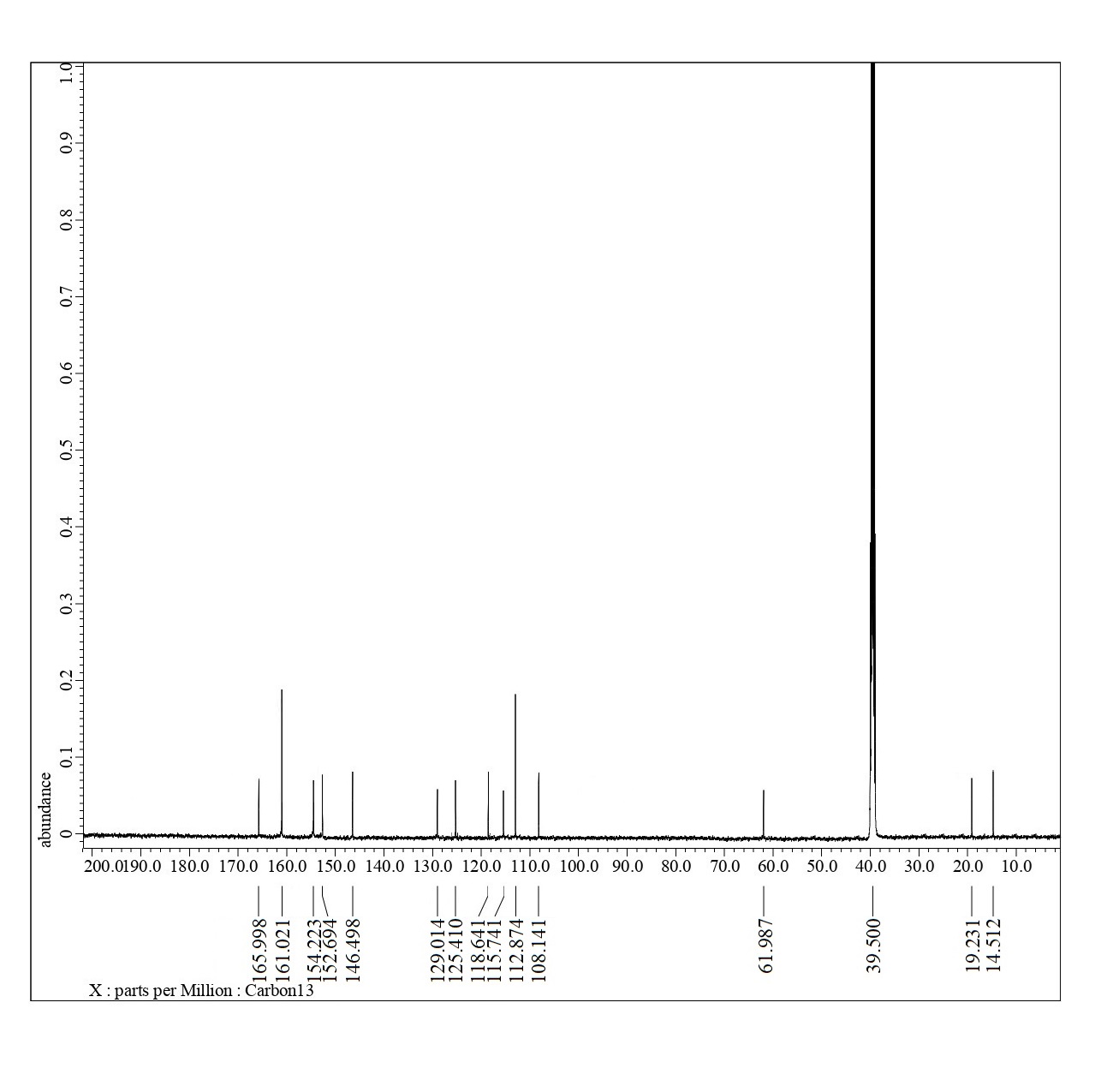
**

**Figure S17.** ^13^C-NMR of compound **16**


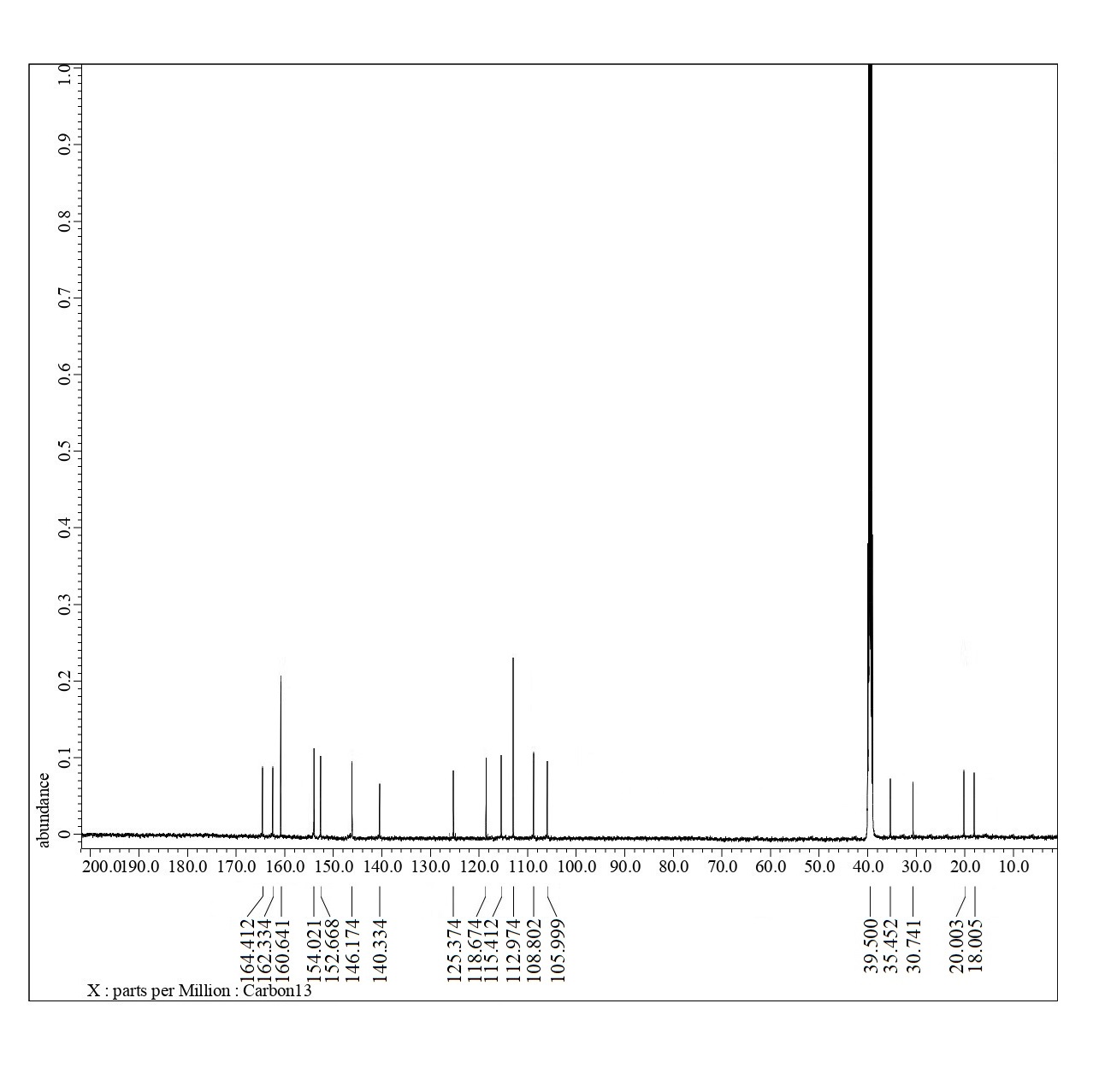


**Figure S18.** ^13^C-NMR of compound **17**


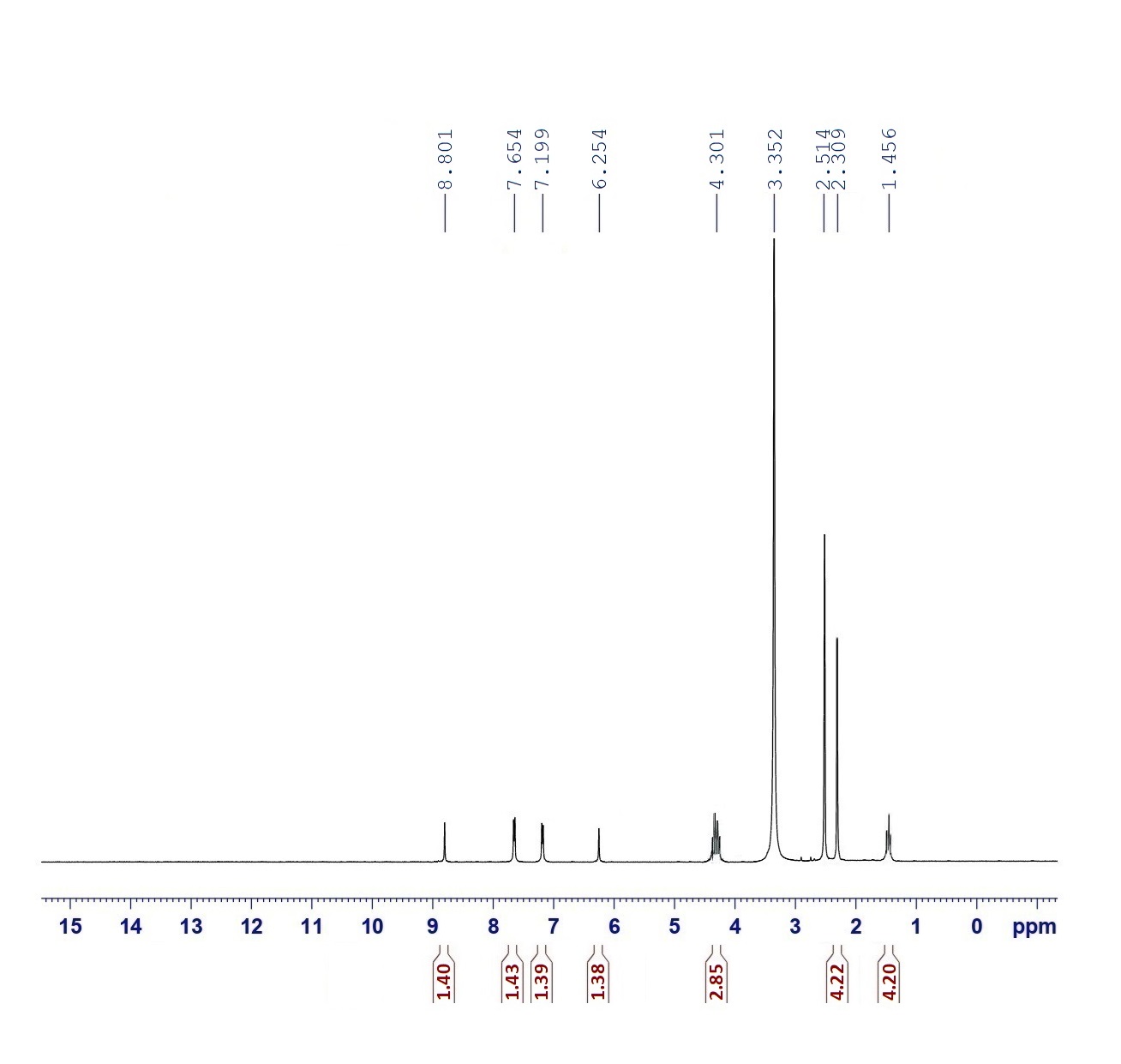


**Figure S19.** ^1^H-NMR of compound **18**
